# Integrated metabolome and transcriptome profiling demonstrates dynamic regulatory roles of hormones in direct-seeding rice

**DOI:** 10.3389/fpls.2026.1767519

**Published:** 2026-03-10

**Authors:** Hang Yang, Zejun Hu, Yong Chen, Xiao Gu, Junhua Ye, Lixia Zhang, Kai Wang, Jianjiang Bai, Liming Cao, Zhenying Shi, Shujun Wu, Ying Yan

**Affiliations:** 1 Key Laboratory of Germplasm Innovation and Genetic Improvement of Grain and Oil Crops (Co-construction by Ministry and Province), Ministry of Agriculture and Rural Affairs, Crop Breeding and Cultivation Research Institute, Shanghai Academy of Agricultural Sciences, Shanghai, China; 2 Shanghai Songjiang District Agricultural Technology Extension Center, Shanghai, China

**Keywords:** CS2022, direct seeding, HXR450, metabolomic, rice, seedling emergence vigor, transcriptomic

## Abstract

**Introduction:**

Direct-seeding rice faces the prominent challenge of low seedling emergence vigor, particularly under deep-sowing mechanical resistance and hypoxic conditions. Although some physiological traits are known, the systemic molecular networks determining superior emergence remain elusive.

**Methods:**

Here, we integrated metabolomic and transcriptomic analyses to compare the elite direct-seeding variety ChongShang2022 (CS2022) with the control Huxiangruan450 (HXR450).

**Results and discussion:**

Weighted gene co-expression network analysis (WGCNA) identified germination-associated metabolic modules. Hub metabolite analysis revealed that the accelerated germination of CS2022 correlates with a higher accumulation of cytokinins (zeatin and *cis*-zeatin-9-N-glucoside), known for antagonizing abscisic acid (ABA)-induced dormancy, alongside key amino acids (e.g., L-lysine) and structural sphingolipids. Physiological validation confirmed the functional significance of these hubs, demonstrating that exogenous *trans*-zeatin and L-lysine significantly promoted seed germination in a dose-dependent manner. Notably, CS2022 exhibited heightened sensitivity, achieving maximal promotion at concentrations approximately 10-fold lower than HXR450. Targeted LC-MS/MS assays further demonstrated that CS2022 maintains a significantly higher GA20/ABA ratio during germination by accumulating the key precursor GA_20_ and deactivating free ABA into ABA-glucosyl ester. This hormonal homeostasis couples with elevated α-amylase activity, accelerating energy mobilization. At the seedling stage, multi-omics integration suggests an optimized growth-defense trade-off in CS2022. Auxin signaling supports rapid elongation, while the upregulation of jasmonic acid (JA) precursor transcripts contrasts with restricted accumulation of bioactive signals (e.g., JA-Ile). This potential signal buffering mechanism likely mitigates growth arrest. Additionally, lipid remodeling involving sphingolipids and waxes may contribute to hypoxia tolerance. Altogether, this study delineates a correlative regulatory network where dynamic hormone buffering, redirected metabolic flux, and adaptive lipid remodeling synergistically maximize direct-seeding rice emergence vigor, providing mechanistic insights and candidate modules for breeding.

## Background

Rice is an important food crop that feeds more than half of the world’s population (Liu et al., 2022). Due to labor shortage and increased agricultural mechanization, direct seeding of rice has been widely adopted in the world (Farooq et al., 2011; Zhou et al., 2019), although problems such as a low whole seedling rate, serious weeds, and easy lodging of rice are still needed to be addressed (Li et al., 2023). Therefore, varieties with high emergence vigor and uniform seedling emergence are better suited to direct seeding. The ability of seedlings to break through deep soil after germination and become established is known as emergence vigor (Mahender et al., 2015). The first priority in choosing direct seeding rice is a high emergence vigor. However, most modern cultivars have poor emergence vigor, resulting in a slow seedling emergence rate and low yields after direct seeding (Li et al., 2023). Deciphering the molecular mechanisms and metabolic basis of emergence vigor could provide theoretical guidance for breeding superior rice varieties with a rapid emergence rate that are suit for direct seeding.

Emergence vigor is mainly determined by seed vigor and seedling vigor (Li et al., 2023). Seed vigor refers primarily to the ability to germinate. This process is regulated by the intricate interplay of amino acid metabolism, hormonal signaling and the metabolism of energy substances, such as starch and sucrose (Rajjou et al., 2012; Zhao et al., 2021). Classically, Gibberellin (GA) and Abscisic Acid (ABA) act antagonistically: germination initiates only when GA signaling overcomes ABA-mediated dormancy. Beyond the classic GA-ABA antagonism, cytokinins also play a pivotal role in seed vigor by promoting cell division and antagonizing ABA-mediated dormancy (Wang et al., 2011). Various internal and external factors regulate the germinating process by affecting the metabolism and signaling of these hormones. For instance, mutations in key genes involved in ABA biosynthesis, such as *OsABA1* and *OsNCED*, which encode zeaxanthin epoxidase and 9-*cis*-epoxycarotenoid dioxygenase respectively, result in pre-harvest sprouting (PHS) (Agrawal et al., 2001). Isopropylmalate synthase (IPMS) is a key enzyme in leucine synthesis. *ipms* mutant exhibits significant reductions in multiple amino acids, GA and the tricarboxylic acid (TCA) cycle, resulting in diminished seed vigor (He et al., 2019). In addition, some other hormones, such as salicylic acid (SA) and brassinosteroid (BR), as well as other factors, such as reactive oxygen species (ROS) also regulate seed vigor by affecting GA metabolism and signaling (He et al., 2024).

High seedling vigor could ensure rapid emergence and early seedling growth to compete with weeds (Rao et al., 2007; Chauhan et al., 2015). To date, many seedling vigor-related QTLs have been genetically identified, but few have been cloned (Zhang et al., 2017). OsGA20 oxidase1 (OsGA20ox1), a key enzyme for gibberellin synthesis, is the first seedling vigor-related factor identified. Variation in its promoter region results in increased GA content as well as elevated seedling vigor (Abe et al., 2012). Recently, Huang et al. discovered that transcription factors OsDREB1A and OsNAC3 form a positive feedback loop through mutual activation, jointly regulating the expression of *OsGA20ox1*, thereby regulating GA biosynthesis and significantly promoting post-germination growth vigor in rice (Huang et al., 2025). Mutation in ent-copalyl diphosphate synthase1 (CPS1), which is also a key enzyme in GA synthesis, results in decreased GA content and reduced seedling vigor (Ma et al., 2022). Mesocotyl length is another important indicator for evaluating seedling vigor. Most genes identified to control mesocotyl length are also related to hormone metabolism (Xiong et al., 2017; Sun et al., 2018; Zhao et al., 2018; Lv et al., 2021; Lyu et al., 2024). Mutation in GAOYAO1 (GY1), the phospholipase for jasmonic acid (JA) synthesis, leads to shorter mesocotyls (Lyu et al., 2024). POLYAMINE OXIDASE 5 (PAO5) is responsible for the synthesis of hydrogen peroxide. Knockdown of *PAO5* results in longer mesocotyls and faster seedling emergence, together with reduced hydrogen peroxide content and increased ethylene content (Lv et al., 2021). Although some of the molecular mechanisms of emergence vigor have been elucidated, the overall transcriptional and metabolic properties of seeds and seedlings during emergence after direct seeding remain poorly understood. Exploring the transcriptional and metabolic profiling of suitable direct-seeding rice varieties would be informative for the corresponding molecular breeding.

Here, we integrated metabolomic and transcriptomic analyses to decipher the superior emergence vigor of the elite direct-seeding variety CS2022 compared to the control HXR450. We identified a coordinated regulatory network where CS2022 exhibits heightened physiological sensitivity to key metabolic hubs (*trans*-zeatin and L-lysine) and maintains a high GA_20_/ABA ratio to accelerate germination. Furthermore, at the seedling stage, we revealed an optimized growth-defense trade-off strategy: CS2022 employs a “signal buffering” mechanism, uncoupling high defense gene transcription from bioactive hormone accumulation, to avoid growth arrest, while simultaneously remodeling lipid metabolism to adapt to hypoxic deep-sowing conditions. This study provides mechanistic insights into the dynamic regulation of emergence vigor and offers potential targets for breeding robust direct-seeding rice.

## Materials and methods

### Plant materials and growth condition

Two *Japonica* rice varieties CS2022 and HXR450 were used in this study. CS2022 exhibited a faster seedling emergence rate, while HXR450 had a relatively slow emergence rate. Both varieties were bred by the Shanghai Academy of Agricultural Sciences. All the rice plants for test were grown in the paddy fields at the experimental station of the Shanghai Academy of Agricultural Sciences in Zhuanghang in summer with routine management.

### Measurement of seed germination rate

Select plump rice seeds and disinfect them in a solution of 15% sodium hypochlorite for 15 minutes. Soak them separately in deionized water or L-lysine solutions at concentrations of 2.5 μM, 25 μM, 250 μM, and 2500 μM, or *trans*-zeatin solution at concentrations of 0.1 mg/L, 0.5 mg/L, 1 mg/L, 5 mg/L and 25 mg/L as required. Then treating them at 35 °C for 36 hours. Subsequently, transfer the seeds to a petri dish lined with two layers of moist filter paper and incubate in a 30 °C constant-temperature incubator. The seeds were considered germinated when either the shoot or the root exceeded half of the length of the seed.

### Measurement of seedling emergence rate, mesocotyl length and plant height

The seeds are dried at 45 °C for three days to break dormancy. Healthy and plump seeds are selected to soak for germination at 35 °C. The uniformly germinated seeds with ~2 mm of coleoptile were evenly sown on the paddy and covered with a layer of 2 cm-thick soil. Then, the planting pots were incubated at 30 °C with 75% humidity for eight days under a 14-h light/10-h dark cycle.

Length of the mesocotyl was measured four days after sowing (DAS). Seedling height was measured continuously from 4 to 8 DAS. Three biological replicates were carried out in each experiment, which contained at least 10 seedlings.

### Untargeted metabonomic profiling

The mature seeds used for the metabolite analysis in this study were harvested in 2024 at maturity. Seeds that had germinated with a coleoptile of approximately 2 mm, as well as seedlings at 5 days after sowing, were collected for metabolite analysis, and six biological repeats were adopted. Metabolite extraction and LC-MS/MS analysis of mature and germinated seeds, as well as seedlings, of CS2022 and HXR450 were performed by Majorbio (China) according to standard procedures. Briefly, 100 mg of the sample was ground into powder using liquid nitrogen. Then, 800 μL of extraction solution (methanol: water = 4:1 (v: v)) containing four internal standards (L-2-chlorophenylalanine (0.02 mg/mL)) was added for metabolite extraction. Samples were ground at 50 Hz for 6 min at -10°C using a frozen tissue grinder. The sample was then extracted by sonication (40 kHz) at 5 °C for 30 minutes, after which it was incubated for a further 30 minutes at -20 °C. The sample was then centrifuged at 4 °C/13,000 g for 15 min, after which the supernatant was transferred to the injection vial for LC-MS/MS analysis. An equal volume of metabolites from all samples was taken and mixed to prepare a quality control (QC) sample. The LC-MS/MS analysis of samples was conducted on a UHPLC-Q Exactive system (Thermo) equipped with an ACQUITY BEH C18 column (100 mm × 2.1 mm i.d., 1.7 μm; Waters, USA). The LC-MS raw data were imported into the metabolomics processing software Progenesis QI (Waters Corporation, Milford, USA) for processing. The processed data were analyzed using the free online majorbio choud platform (cloud.majorbio.com). To identify differentially accumulated metabolites, OPLS-DA was applied. Metabolites with a VIP value greater than 1, indicating an above-average contribution to group discrimination and robustness to a moderate sample size, were retained. These were then filtered using a student’s t-test (*p* < 0.05) to balance statistical power and control for false positives in discovery-oriented profiling. Only metabolites that met both criteria were considered significant.

### Weighted gene co-expression network analysis of the metabolites

To further investigate the accumulation patterns of metabolites during seedling emergence, we constructed co-accumulation network modules using WGCNA (Langfelder and Horvath, 2008). First, the obtained non-targeted metabolomics data underwent rigorous preprocessing. Missing values were handled according to the following rules: if the missing rate of a metabolite exceeded 50% in any experimental group, it was excluded; for other random missing values, the metabolite’s minimum abundance within the same group was used to fill in half of the missing values. Subsequently, data underwent sum normalization to correct for loading variations between samples and were log2-transformed to approximate a normal distribution. Finally, each metabolite underwent unit variance scaling to achieve a mean of 0 and standard deviation of 1, eliminating dimensionality effects. Based on the preprocessed metabolite abundance matrix, we constructed a co-accumulation network using the WGCNA R package. By analyzing network topology under different soft threshold powers, we selected power *β* = 7 to ensure the constructed network exhibited scale-free distribution characteristics (scale-free topological fit R² > 0.85). Based on this, we computed the topological overlap matrix among metabolites and employed the dynamic mixed-tree cutting algorithm for hierarchical clustering to identify co-accumulation modules. The minimum module size was set to 30 metabolites, and highly similar modules (feature vector correlation > 0.75) were merged.

To identify metabolic modules associated with seedling emergence, samples from both varieties were separately categorized into three stages: mature seed, germinating seed, and seedling stage. Pearson correlations between each module’s eigenvector and the trait were calculated, yielding statistically significant *P* values. Modules significantly correlated with the trait (*P* value < 0.05) were selected for subsequent analysis. For key modules, we extracted their module membership (MM) and gene significance (GS) values, and further identified hub metabolites within each module (i.e., metabolites with the highest connectivity within the module). The hub metabolites network is displayed by Cytoscape (Shannon et al., 2003).

### Phytohormone detection

The contents of phytohormones were detected by MetWare (http://www.metware.cn/) using the AB Sciex QTRAP 6500 LC-MS/MS platform. Phytohormones were extracted from rice seeds that were germinating and flash-frozen in liquid nitrogen, three biological repeats were adopted for detection. Approximately 50 mg of the resulting powder was then homogenized in 1 ml of a methanol/water/formic acid solution (15:4:1, v/v/v), to which internal standards were added. Following vertexing and centrifugation, the resulting supernatant was dried under nitrogen, reconstituted in 80% methanol and filtered through a 0.22 µm membrane. Analysis was performed on a UPLC-ESI-MS/MS system. Separation was achieved using a Waters ACQUITY UPLC HSS T3 C18 column with a gradient of water and acetonitrile, both of which contained 0.04% acetic acid. Mass spectrometry detection was conducted in scheduled MRM mode under optimized positive/negative electrospray ionization conditions. Compounds were quantified using internal standard methods by comparing their MRM transitions and retention times to those of authentic standards.

### Determination of amylase and *α*-amylase activities

The activities of amylase and *α*-amylase were determined by measuring reducing sugars released from soluble starch using the 3,5-Dinitrosalicylic acid (DNS) method. Frozen germinating seeds (0.5 g) were homogenized in ice-cold 20 mM phosphate buffer (pH 6.9, containing 100 mM NaCl and 1 mM CaCl_2_) and centrifuged at 12,000 × g for 20 min at 4 °C to obtain the crude enzyme extract. For total amylase activity, 100 µL of extract was incubated with 900 µL of 1% soluble starch at 37 °C for 10 min. For *α*-amylase activity, an aliquot of the extract was first incubated at 70 °C for 15 min to inactivate *β*-amylase before the assay. The reaction was terminated by adding DNS reagent, followed by boiling for 5 min. After cooling, the absorbance was measured at 540 nm. Enzyme activity was calculated using a maltose standard curve.

### RNA extraction, library preparation, sequencing and data analysis

Total RNA was extracted from seedlings using TRIzol^®^ Reagent, following the manufacturer’s instructions. The quality of the RNA was determined using a 5300 Bioanalyser (Agilent), and the concentration was determined using an ND-2000 (NanoDrop Technologies). High-quality RNA (OD260/280 = 1.8–2.2, OD260/230 ≥ 2.0, RQN ≥ 6.5, 28S:18S ≥ 1.0, >1 μg) was then used for library construction, after which high-throughput sequencing was performed on the NovaSeq X Plus platform. Three biological repeats were used. After filtering, about 6.5 Gb of clean data was obtained for each sample. These clean data were then compared with the reference genome (IRGSP-1.0) to obtain mapped data for subsequent transcript assembly, expression calculation, etc. Differential expression analysis was performed using DESeq2. Genes with FDR < 0.05 (Benjamini-Hochberg) were deemed statistically significant, controlling for multiple testing. An additional filter of |log_2_ FC| ≥ 1 was applied to capture physiologically relevant expression shifts, a threshold widely adopted in rice seed transcriptome studies. Genes satisfying both criteria were defined as differentially expressed. DEGs were annotated according to Gene Ontology (GO) (http://geneontology.org/) for their participation in Biological Process (BP), Cellular Component (CC) and Molecular Function (MF). The KEGG database was used to categorize genes according to the pathways they participate in or the functions they perform (https://www.genome.jp/kegg/).

### Quantitative real-time PCR

Eight DEGs were selected for validation of the RNA-seq data using qRT-PCR. Total RNA was extracted from seedlings 5 DAS and reverse-transcribed according to the manufacturer’s instruction. The primers used for qRT-PCR were designed using the NCBI Primer BLAST program (https://www.ncbi.nlm.nih.gov/tools/primer-blast/index.cgi?LINK_LOC=BlastHome) (**Supplementary Table 11**). qRT-PCR was performed using an ABI 7500 Real-Time PCR System, with *UBQUTIN* used as the internal reference gene. Each sample contained three biological replicates and technical replicates. The relative expression of the target genes was calculated using the 2^-ΔCt^ formula.

## Results

### The CS2022 variety exhibits higher seed vigor and seedling vigor compared to HXR450

**Figure 1:**
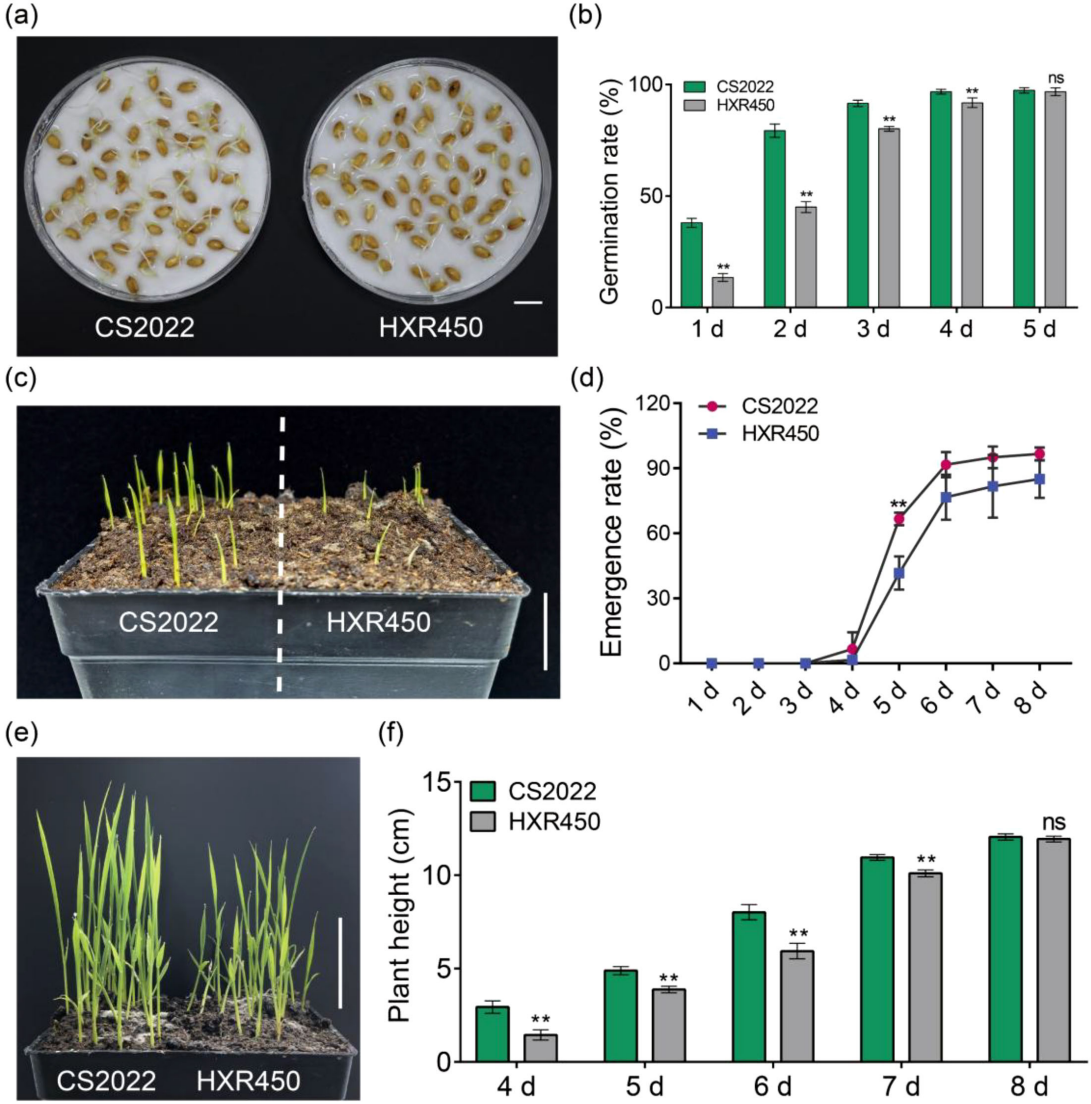
Germination, seedling emergence rate and seedling vigor of CS2022 and HXR450. **(A)** Germination performance of CS2022 and HXR450 seeds two days after germination induction. Scale bar = 1 cm. **(B)** The statistics of germination rates of CS2022 and HXR450 seeds 1 to 5 days after germination induction. Each sample comprised three biological replicates, with 50 seeds per replicate. Data are mean ± SD. Student’s *t*-test was carried out (**P < 0.01). **(C)** The status of seedling emergence of CS2022 and HXR450–5 DAS at 2 cm below the soil. Scale bar = 2.5 cm. **(D)** The statistics of emergence rate of CS2022 and HXR450 within 8 DAS. Data are mean ± SD (n = 3). Student’s *t*-test was carried out (**P < 0.01). **(E)** The status of CS2022 and HXR450 seedling 6 DAS. Scale bar = 3 cm. **(F)** The statistics of seedling height 4–8 DAS. Data are mean ± SD (n = 10). Student’s *t*-test was carried out (**P < 0.01).

CS2022 and HXR450 are *Japonica* rice varieties cultivated by ourselves, which are distinguished in their high-quality, especially excellent eating and cooking quality (ECQ). However, they differ significantly in terms of direct-seeding properties. Specifically, the CS2022 seeds germinated faster and exhibited more uniform germination than HXR450 seeds (**Figures 1A, B**). Two days after germination induction, the germination rate of CS2022 seeds reached ~80%, while that of HXR450 seeds was only around 45%. This trend of higher germinating rate kept well along with the germinating process (**Figure 1B**). Meanwhile, CS2022 seeds showed significantly faster shoot and root growth compared to HXR450 seeds (**Supplementary Figure 1A**). Moreover, the two varieties also showed significant differences in early post-germination growth. When uniformly germinating seeds were sown 2 cm below the soil, the seedlings of CS2022 emerged much faster than those of HXR450 (**Figure 1C**). Five days after sowing (DAS), more than 65% of CS2022 seedlings emerged from the soil, while less than 40% of HXR450 seedlings emerged. During the whole germinating process, the seedling emergence rate of HXR450 was consistently lower than that of CS2022 (**Figure 1D**). Mesocotyl elongation is an important characteristic of suitable direct-seeding rice varieties (Li et al., 2023). However, there is no significant difference in mesocotyl length (the distance from cotyledon to coleoptile) between CS2022 and HXR450 (**Supplementary Figures 1B, C**). Further continuous observation revealed that CS2022 grew much faster than HXR450 (**Figure 1E**), with higher plant height at early seedling growth stage (**Figure 1F**). But little difference in plant height was observed between the two varieties at 8 DAS (**Figure 1F**). The above results suggested that the faster and more uniform germination, as well as quicker emergence and growth of CS2022 at early seedling stage makes it more suitable for direct seeding.

### Metabolite profiles of seeds and seedlings during rice seedling emergence

**Figure 2:**
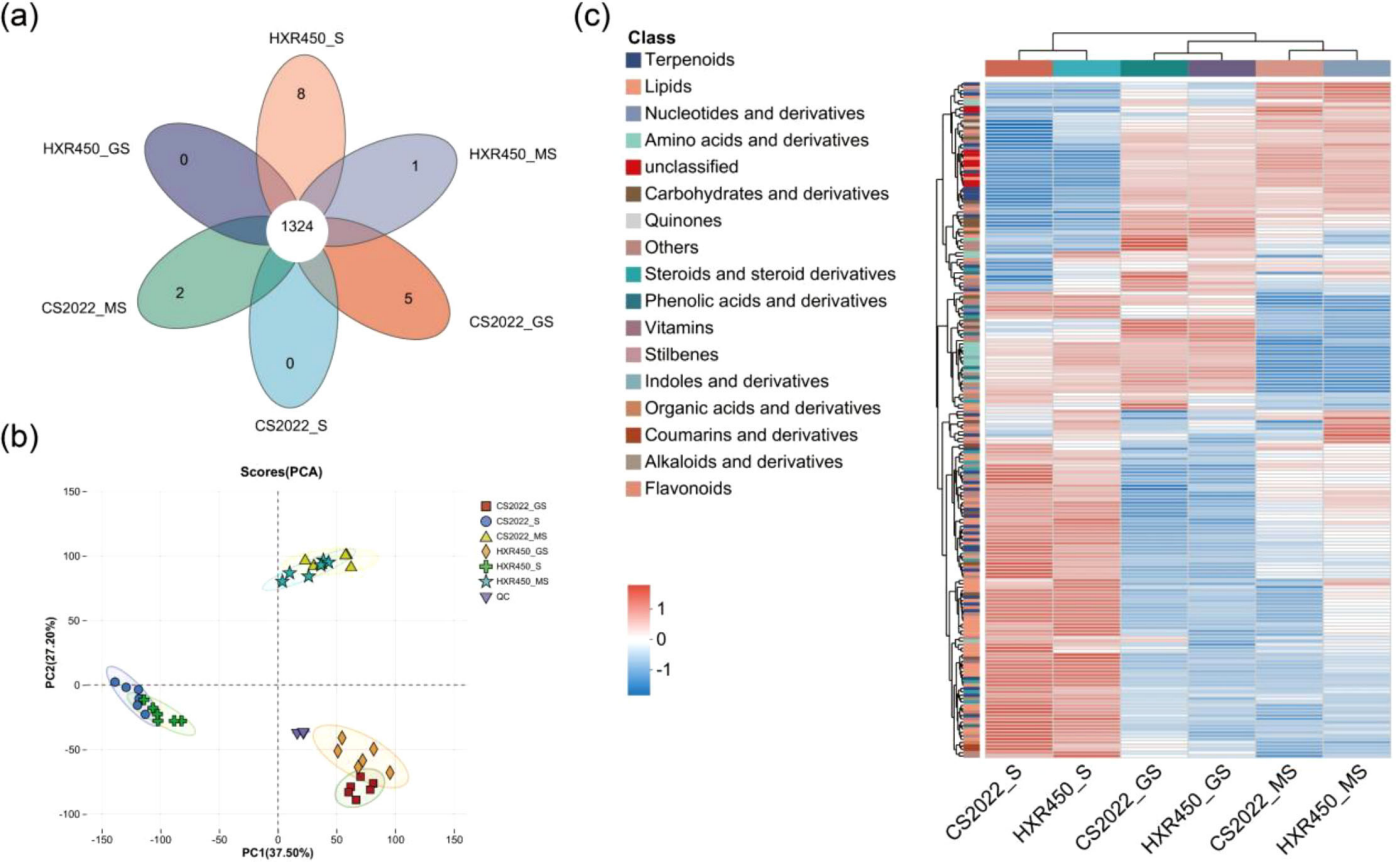
Metabolic variation in CS2022 and HXR450 seeds and seedlings at different stages. **(A)** Venn diagram of metabolites detected in the three stages of CS2022 and HXR450. **(B)** Principal component analysis (PCA) of metabolites profiling of CS2022 and HXR450 mature seeds (MS), germinating seeds (GS) and seedlings (S). Six replicates were used per sample. **(C)** The heatmap of metabolites in the three stages of CS2022 and HXR450 during seedling emergence. Hierarchical cluster of metabolite species was also performed. Six replicates were used per sample. Columns represent samples and rows represent metabolites. Red indicates higher metabolite abundance, while blue indicates lower metabolite abundance as indicated by the scalebar.

To identify the active metabolic pathways during different stages, as well as the metabolomic differences that may account for the variation in germination and emergence rates between CS2022 and HXR450, we measured the content of metabolites in mature seeds (MS), germinating seeds (GS) and seedlings (S) of these two varieties. As demonstrated, 912 metabolites were identified in positive ion mode (POS), and 522 metabolites in negative ion mode (NEG). Among them, 1,324 metabolites were found in all samples (**Figure 2A**, **Supplementary Table 1**). Principal component analysis (PCA) showed that the samples were highly repeatable and significantly separated at different stages, suggesting that they could be used for further metabolomic analysis. Meanwhile, the PCA score plot showed that the separation between the different stages is much greater than the separation between varieties (**Figure 2B**). Therefore, metabolic variation at different stages is much more than that between varieties at the same stage. Also, heatmap analysis of metabolites in CS2022 and HXR450 at different stages confirmed such a tendency (**Figure 2C**). For example, germinating seeds and seedlings had significantly higher amino acids and derivatives abundance relative to mature seeds. In addition, the metabolite profiles between the two varieties at the same stage were also significantly different. Some amino acids and derivatives were more abundant in CS2022 than in HXR450 seedlings (**Figure 2C**, **Supplementary Table 1**). Such as O-Acetylserine, an intermediate in the synthesis of cysteine. In conclusion, the metabolite profiles of mature seeds, germinating seeds and newly emerged seedlings varied considerably, indicating that distinct key metabolites might be motivated to exert significant influence at different stages of direct-seeding. In addition, the abundance of certain metabolites varied significantly between the CS2022 and HXR450 cultivars, which might be the underlying reason for the advantageous properties of CS2022 in the direct-seeding process.

### Identifying metabolic modules correlated with germination and post-germination growth by WGCNA

**Figure 3:**
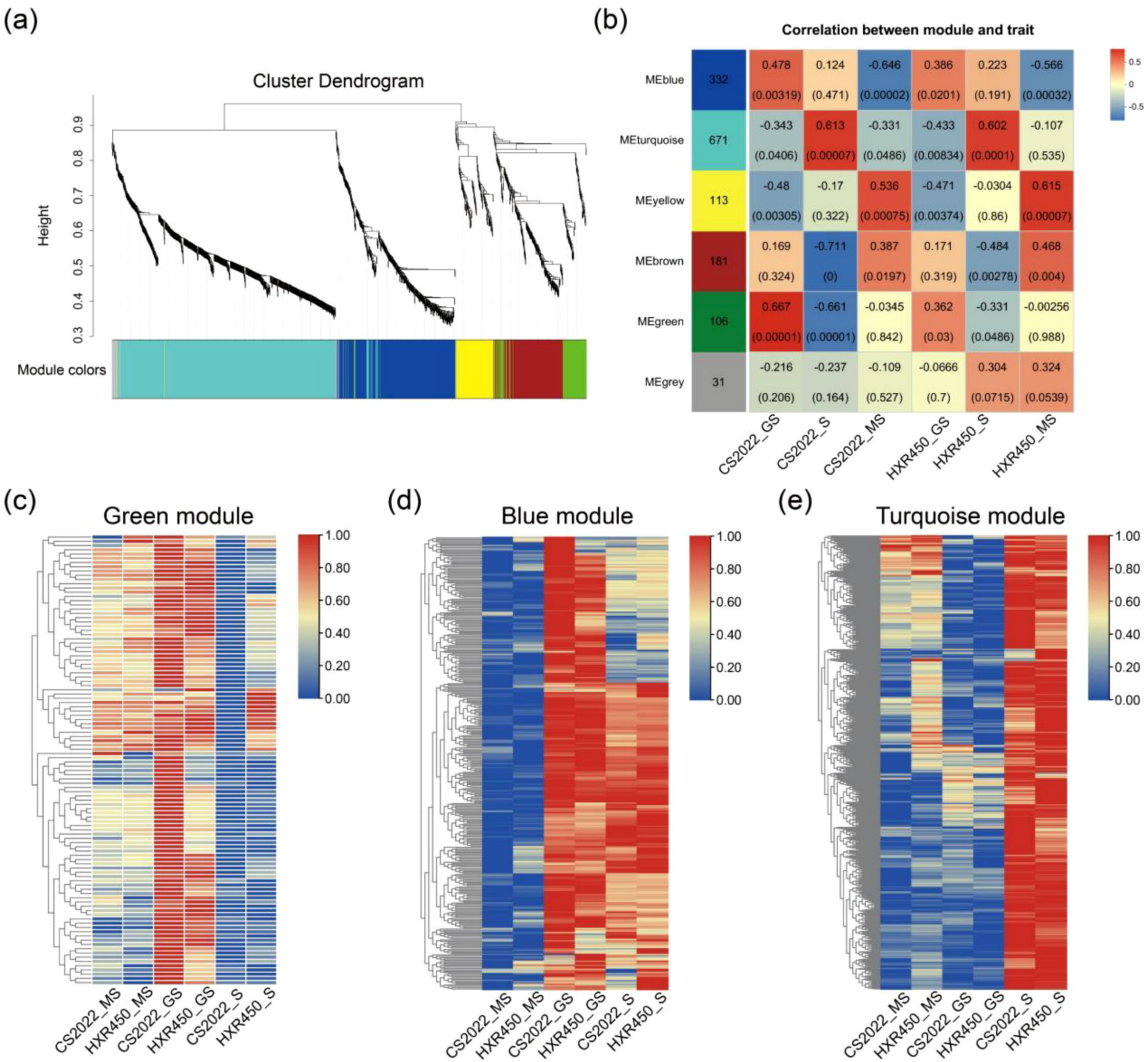
Key metabolomic modules associated with seedling emergence obtained by WGCNA. **(A)** Cluster dendrogram showing 5 modules of co-accumulation metabolites identified by WGCNA. The major tree branches constitute 5 modules, labeled with different colors. **(B)** Relationship between modules and different developmental stages of direct seeding. The horizontal axis represents different stages, while the vertical axis represents different modules. The left column of numbers indicates the number of metabolites for each module, and the right column shows the correlation coefficient between the modules and stages along with the significance *P* value in parentheses. Higher absolute values indicate stronger correlations, with red denoting positive correlations and blue indicating negative correlations. **(C–E)** Heatmap of metabolites within three modules. Data has been normalized. Red color indicates higher abundance, while blue indicates relatively lower abundance.

We further conducted WGCNA to systematically decipher the pattern of metabolite accumulation and identify the difference in metabolic regulation between the control variety HXR450 and the direct-seeding variety CS2022. A total of 6 distinct modules were identified according to the similar co-accumulation trends of three stages (**Figure 3A**). The number of metabolisms per module ranged from 31 (grey) to 671 (turquoise). Pearson correlation analysis between modules and the three stages of direct-seeding (mature seeds, germinating seeds and seedlings) indicated that the green and blue modules exhibited a significantly positive correlation with germinating seeds (*P* < 0.05), the turquoise module showed a positive correlation with the seedling stage (*P* < 0.01), and the yellow and brown modules are positively correlated with mature seeds (*P* < 0.05) (**Figure 3B**). We intended to investigate the specific accumulation of metabolites during the germination and seedling stages and their potential roles in rapid seedling emergence. Therefore, we conducted an in-depth analysis of the green, blue, and turquoise modules.

### Identification of germination related metabolic modules

**Figure 4:**
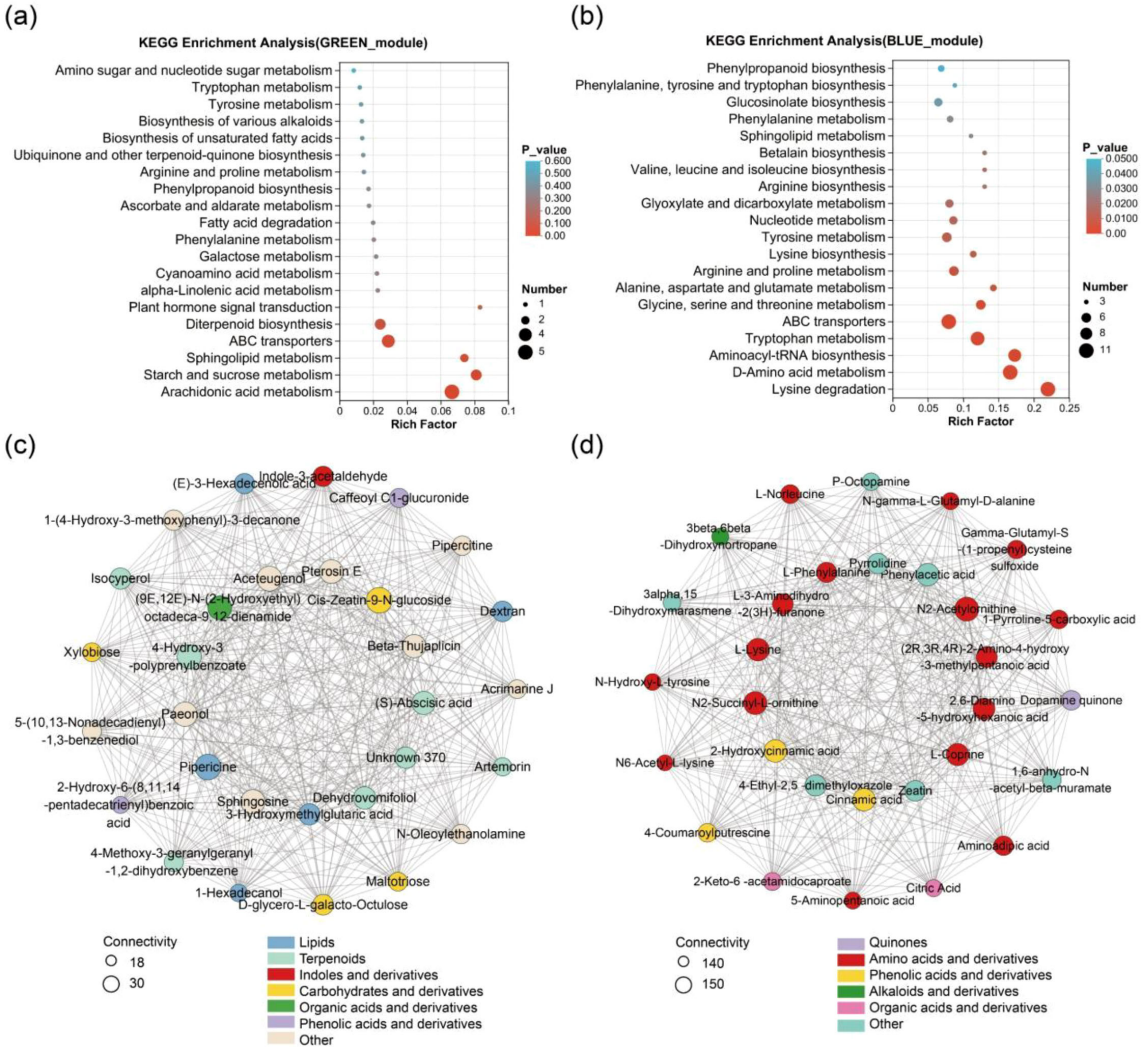
KEGG enrichment analysis, and hub metabolites correlation network diagram of green and blue modules. **(A, B)** The KEGG enrichment analysis of the green **(A)** and blue **(B)** modules. A larger rich factor indicates a greater degree of enrichment. The size of the dots indicates the number of metabolites in the pathway, and the color of the dots corresponds to different *P* value ranges. **(C, D)** The correlation network of hub metabolites identified from the green **(C)** and blue **(D)** modules. Larger nodes indicate greater connectivity of metabolites. Node colors denote different compound categories.

The green and blue modules contain 106 and 332 metabolites, respectively (**Figure 3B**, **Supplementary Table 2**). In both CS2022 and HXR450, the two modules exhibited high metabolite abundance in germinating seeds (**Figures 3C, D**). Some metabolites in the blue module also showed high accumulation at the seedling stage (**Figure 3D**). Metabolites in the green module were significantly enriched in pathways including arachidonic acid metabolism, starch and sucrose metabolism, sphingolipid metabolism, ABC transporters, diterpene biosynthesis, and plant hormone signal transduction (**Figure 4A**, **Supplementary Table 3**). Metabolites in the blue module were significantly enriched in various amino acid metabolic pathways, including key amino acids such as lysine, tryptophan, phenylalanine and D-amino acids (**Figure 4B**, **Supplementary Table 3**). Blue module was also significantly enriched in pathways related to translation, substance transport, lipid metabolism, and carbohydrate metabolism, including aminoacyl-tRNA biosynthesis, nucleotide metabolism, ABC transporters, glyoxylate and dicarboxylate metabolism, and sphingolipid metabolism. The roles of hormones and carbohydrate metabolism in rice seed germination have been extensively studied (Rajjou et al., 2012; Xiong et al., 2022). Our findings indicated that lipids such as sphingolipids and arachidonic acid, along with fundamental substances like nucleotide and amino acids, also exhibited high abundance during rice germination, suggesting that these metabolisms might play a positive role in the germination process.

To obtain key metabolic factors within the aforementioned pathways, the top 30 metabolites with the highest connectivity in the green and blue modules were screened to construct a hub metabolites correlation network respectively (**Figures 4C, D**, **Supplementary Table 4**). It is found that certain carbohydrates, such as Dextran, D-glycero-L-galacto-Octulose, Xylobiose, Maltotriose and *Cis*-Zeatin-9-N-glucoside, the Indole acetic acid (IAA) precursor indole-3-acetaldehyde, (S)-abscisic acid and sphingosine exhibited high connectivity within the green module (**Figure 4C**). In the blue module, 17 of the top 30 metabolites with highest connectivity were amino acids and their derivatives, including L-lysine, L-phenylalanine, and the cytokinin zeatin. Additionally, other highly correlated metabolites in the blue module were also associated with amino acid metabolism. For example, the organic acid L-norleucine is a degradation product of lysine (**Figure 4D**). Therefore, it is highly possible that metabolites in amino acid metabolism play a critical role in rice germination.

### The difference in the accumulation of germination-related hub metabolites between CS2022 and HXR450

**Figure 5:**
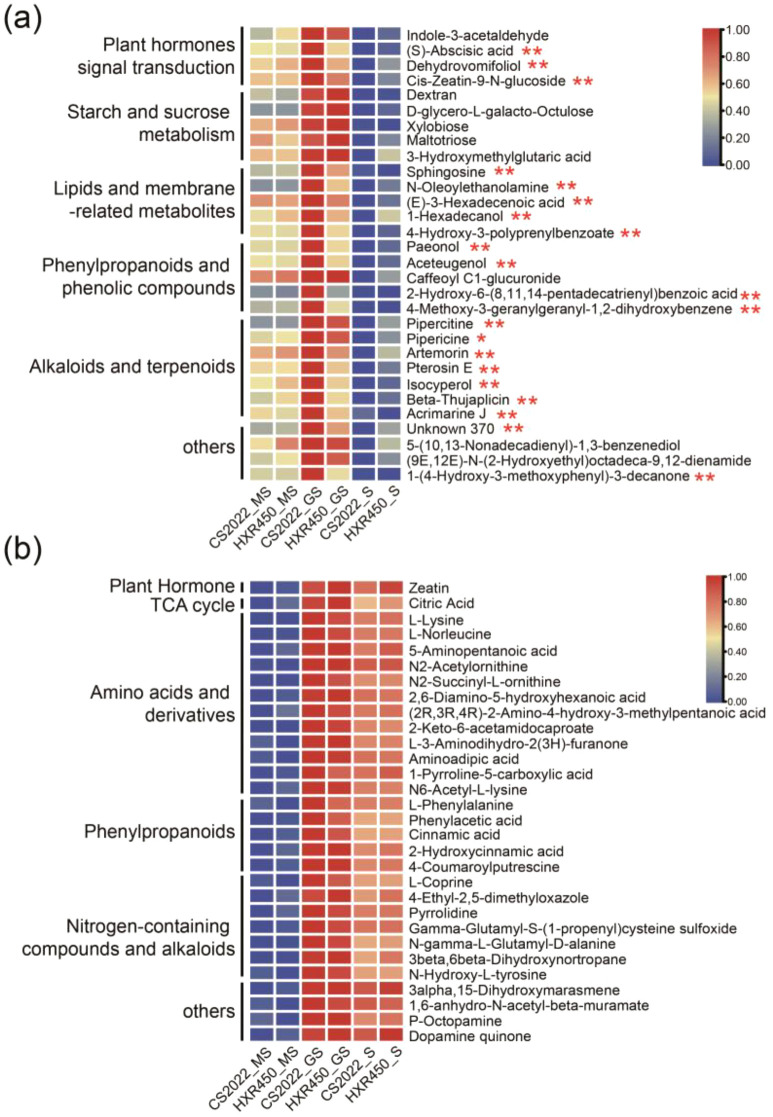
Heatmap of hub metabolites in the green and blue modules of CS2022 and HXR450. **(A, B)** Heatmap of the 30 hub metabolites identified in the green **(A)** and blue **(B)** modules. Metabolites are annotated according to their pathways. Data has been normalized. Student’s t-test was carried out (*P < 0.05, **P < 0.01).

To further investigate the mechanism underlying the faster germination rate of CS2022, we compared the abundance of these hub metabolites between CS2022 and HXR450 (**Figures 5A, B**, **Supplementary Table 4**). In the green module, there was no significant difference between the two varieties in terms of starch and sucrose metabolites (maltotriose, dextran, xylobiose, D-glycero-L-galacto-octulose). However, germinating seeds of the two varieties exhibited distinct difference in the levels of certain plant hormones. Compared to HXR450, CS2022 germinating seeds contained higher levels of *Cis*-Zeatin-9-N-glucoside, a storage form of cytokinin. At the same time, CS2022 germinated seeds contained slightly higher levels of abscisic acid than HXR450. Additionally, certain lipids and membrane-related metabolites, such as Sphingosine, N-Oleoylethanolamine, 1-Hexadecanol, along with phenylpropanoids and phenolic compounds, such as Aceteugenol, Paeonol, 4-Methoxy-3-geranylgeranyl-1,2-dihydroxybenzene and 4-Hydroxy-3-polyprenylbenzoate exhibited relatively high abundance in CS2022 germinating seeds (**Figure 5A**). These metabolites, respectively implicated in lipid signaling (Berkey et al., 2012; Zhou and Ye, 2022), membrane stability (Teaster et al., 2007), and antioxidant activity (Dixon and Paiva, 1995), may contribute to the enhanced seed vigor of CS2022.

Meanwhile, in the blue module, the 30 filtered metabolites with high connectivity showed significantly higher accumulation in germinating seeds and seedlings compared to in mature seeds (**Figure 5B**). These hub metabolites include zeatin, citric acid, certain amino acids and their derivatives, phenylpropanoid metabolites, and others. The cytokinin zeatin and the TCA cycle both promote germination and growth (Yuan et al., 2008; Sweetlove et al., 2010; Farooq et al., 2021), while phenylpropanoid compounds have been reported to play a crucial role in defense against diseases and pests (Dixon and Paiva, 1995). However, no significant differences were observed between CS2022 and HXR450 (**Figure 5B**, **Supplementary Table 4**), indicating these metabolites are generally needed for germination and preliminary growth.

Simultaneously, we performed differential accumulation analysis and KEGG analysis on metabolites in germinating seeds of CS2022 and HXR450 (**Supplementary Table 5**, **6**). The levels of 303 and 87 metabolites were respectively increased and decreased in the germinating seeds of CS2022 as compared to HXR450 (**Supplementary Figure 2A**). The altered metabolites were primarily enriched in lipid metabolism, amino acid metabolism, and plant hormone signal transduction pathways. Moreover, most of the metabolites were up-regulated (**Supplementary Figure 2B**). These DAM-enriched pathways largely overlap with those enriched by green and blue modules, with significant overlap in the involved metabolites (**Supplementary Figures 2C, D**). Notably, over half of the metabolites in the green module represented DAMs between CS2022 and HXR450 germinating seeds, with the vast majority being enhanced (**Supplementary Figure 2C**).

In summary, CS2022 seeds exhibited higher levels of hormones such as zeatin, lipids like sphingolipids, certain phenylpropanoid compounds, and key amino acids like L-lysine during the germinating process, which might account for its enhanced seed vigor.

### Elevated GA precursors and decreased ABA contribute to rapid germination in CS2022

**Figure 6:**
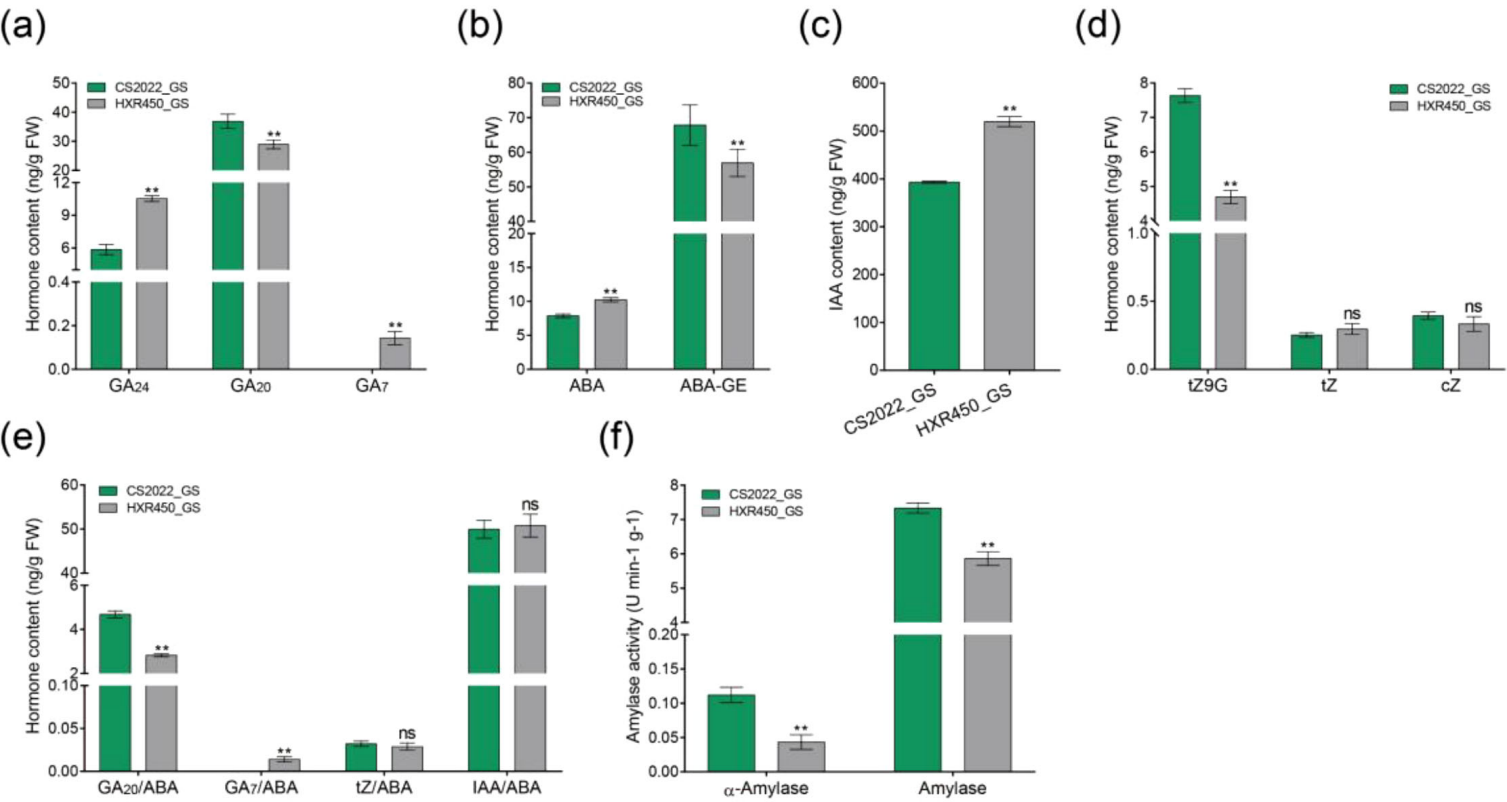
Endogenous hormone levels and amylase activities in CS2022 and HXR450 germinating seeds. **(A)** Gibberellins (GA_24_, GA_20_, and GA_7_) content in CS2022 and HXR450. **(B)** Content of abscisic acid (ABA) and its inactivated conjugate ABA-glucosyl ester (ABA-GE) in CS2022 and HXR450. **(C)** Indole-3-acetic acid (IAA) content in CS2022 and HXR450. **(D)** Content of cytokinin metabolites: *trans*-Zeatin-9-glucoside (tZ9G), *trans*-Zeatin (tZ), and *cis*-Zeatin (cZ) in CS2022 and HXR450. **(E)** Ratios of key hormones (GA_20_/ABA, GA_7_/ABA, tZ/ABA, and IAA/ABA) in CS2022 and HXR450. **(F)** Activities of *α*-amylase and total amylase in CS2022 and HXR450. All data were got through measuring in germinating seeds (GS) of CS2022 and HXR450. Data represent mean ± SD (n = 3). Asterisks indicate significant differences between the two varieties determined by Student’s t-test (** *P* < 0.01; ns, not significant).

To validate the physiological mechanisms underlying the superior vigor of CS2022 seeds, we quantified key phytohormones using targeted LC-MS/MS analysis and measured amylase activities (**Figure 6**, **Supplementary Table 7**). This targeted approach revealed distinct profiles for gibberellins (GAs), abscisic acid (ABA), cytokinins, and auxin between the two varieties.

Regarding gibberellin metabolism, three primary forms were detected: the bioactive GA_7_, the key precursor GA_20_, and the upstream precursor GA_24_ (Huang et al., 2025). While the content of GA_7_ and GA_24_ was significantly higher in HXR450 germinating seeds, CS2022 exhibited a substantial accumulation of GA_20_ (**Figure 6A**). Crucially, the targeted analysis clarified the ABA profile: CS2022 maintained significantly lower levels of free ABA and higher levels of the inactive conjugate ABA-glucosyl ester (ABA-GE) compared to HXR450 (**Figure 6B**). This resolves the ambiguity from our previous untargeted metabolomics, which could not distinguish between active and inactive forms, previously suggesting slightly higher total ABA pools in CS2022. The balance between GA and ABA is a critical determinant of germination. In CS2022, the GA_20_/ABA ratio was nearly double that of HXR450, although the GA_7_/ABA ratio remained lower (**Figure 6E**). GA_20_ is the immediate precursor of the bioactive GA_1_/GA _3_ (Huang et al., 2025). CS2022 accumulated significantly higher levels of GA_20_, indicating a robust biosynthetic flux ready to drive germination. The HXR450 control variety exhibits a bottleneck at the GA_24_ conversion step, whereas the CS2022 variety efficiently converts it into GA_20_. We propose that the elevated pool of GA_20_, coupled with effective ABA deactivation, facilitates the accelerated germination observed in CS2022. Regarding other hormones, CS2022 displayed lower auxin levels, though the IAA/ABA ratio showed no significant difference (**Figure 6C**). Furthermore, while the active cytokinin *trans*-Zeatin showed no variation, significant differences were observed in its storage form, *trans*-Zeatin-9-glucoside, between the varieties (**Figure 6D**).

Consistently, CS2022 exhibited significantly enhanced *α*-amylase and total amylase activities (**Figure 6F**), indicating a more efficient mobilization of starch reserves to fuel rapid growth.

### Exogenous *trans*-Zeatin and L-Lysine differentially regulate seed germination

**Figure 7:**
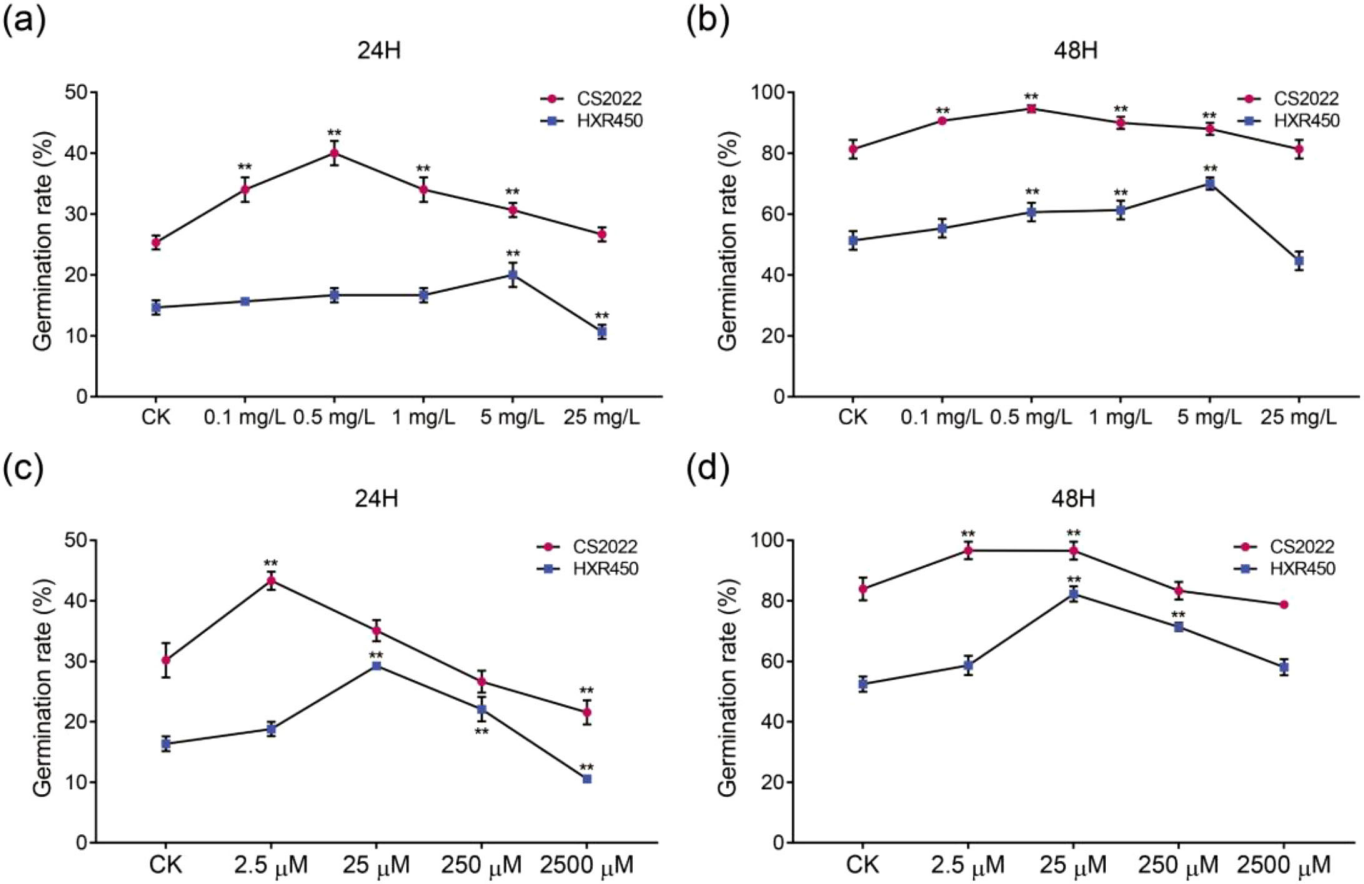
Effects of exogenous *trans*-zeatin and L-lysine treatments on seed germination of CS2022 and HXR450. **(A, B)** Germination rates of CS2022 and HXR450 seeds treated with different concentrations of *trans*-zeatin (0, 0.1, 0.5, 1, 5, and 25 mg/L) at 24 h **(A)** and 48 h **(B)**. **(C, D)** Germination rates of CS2022 and HXR450 seeds treated with different concentrations of L-lysine (0, 2.5, 25, 250, and 2500 µM) at 24 h **(C)** and 48 h **(D)**. Data represent mean ± SD (n = 3). Asterisks indicate significant differences compared to the control (CK) for each variety (** *P* < 0.01).

Our previous WGCNA analysis identified zeatin and L-lysine as central hub metabolites within the germination-associated blue module (**Figures 4**, **5**), and targeted LC-MS/MS assays confirmed significant variances in cytokinin metabolites (e.g., *trans*-Zeatin-9-glucoside) between the two varieties (**Figure 6D**). To verify the biological functions of these hubs and explore varietal sensitivity, we performed exogenous priming experiments on CS2022 and HXR450 seeds (**Figure 7**). Guided by established roles of cytokinin in dormancy release (Wang et al., 2011; Guan et al., 2014) and amino acid signaling (Galili, 2002; Galili and Amir, 2013; He and Yang, 2013), we applied concentration gradients of *trans*-zeatin (tZ) (0.1–25 mg/L) and L-lysine (2.5-2500 µM).

Exogenous *trans*-zeatin significantly promoted germination in both varieties, yet they displayed distinct sensitivity thresholds (**Figures 7A, B**). The high-vigor variety CS2022 exhibited a hypersensitive response, with germination rates peaking at a concentration of 0.5 mg/L. At this dosage, germination at 24 hours increased from ~25% (Control) to ~40%, reaching ~95% by 48 hours. Notably, higher concentrations (25 mg/L) inhibited germination in CS2022, consistent with a hormetic dose-response. In contrast, the low-vigor variety HXR450 displayed a “hyposensitive” response, requiring a ten-fold higher concentration (5 mg/L) to achieve maximum promotion at both 24h and 48h. These results suggest that CS2022 operates with a lower, more active physiological threshold for cytokinin signaling, whereas HXR450 requires higher exogenous supplementation to overcome internal inhibition, potentially imposed by ABA.

The other identified metabolic hub, L-lysine, exhibited a similar hormetic dose-response: low concentrations promoted germination while high concentrations proved inhibitory (**Figures 7C, D**). In Parallel, the hormone trials, CS2022 was more sensitive, achieving optimal germination promotion at 2.5 µM (rising to ~43% germination at 24h). Conversely, HXR450 required 25 µM L-lysine to reach its peak rate (~30% at 24h). Excessive L-lysine (2500 µM) significantly inhibited germination in both varieties. Collectively, these findings validate that the hub metabolites identified by WGCNA are functionally active regulators of germination, and the superior vigor of CS2022 is intrinsically linked to its higher physiological sensitivity to these metabolic cues.

### Identification of seedling-establishment related metabolic module

**Figure 8:**
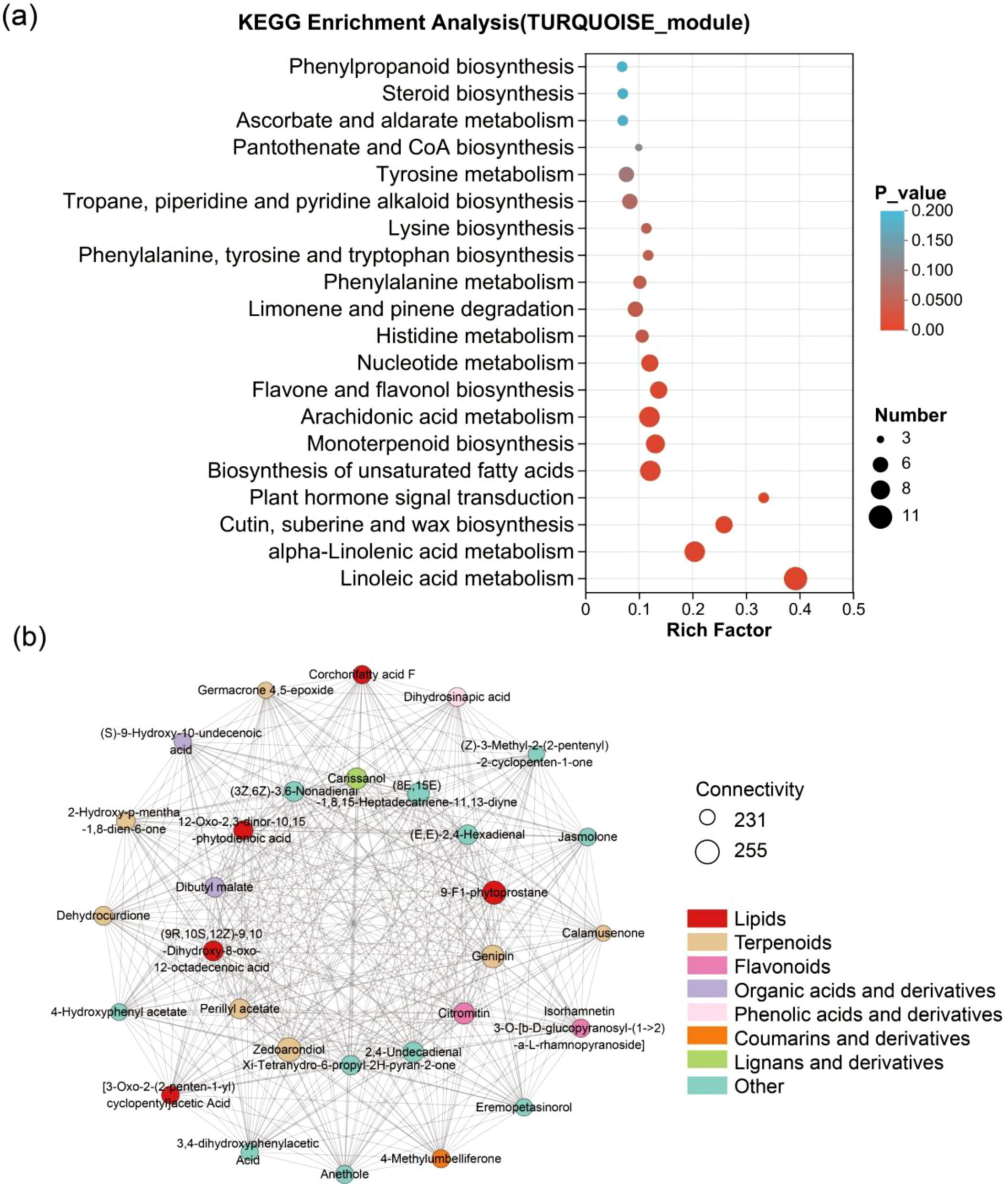
KEGG enrichment analysis and hub metabolites correlation network diagram of the turquoise module. **(A)** The KEGG enrichment analysis of the turquoise module. A larger rich factor indicates a greater degree of enrichment. The size of the dots indicates the number of metabolites in the pathway, and the color of the dots corresponds to different *P* value ranges. **(B)** The correlation network of hub metabolites identified in the turquoise module. Larger nodes indicate greater connectivity of metabolites. Node colors denote different compound categories.

The turquoise module, which was related with the seedling stage comprised 671 metabolites, whose abundance is significantly higher during the seedling stage than in mature seeds and germinating seeds (**Figures 3B, E**, **Supplementary Table 2**). KEGG pathway enrichment analysis of metabolites from this module revealed significant enrichment in multiple pathways associated with lipid metabolism, plant hormone signaling, and secondary metabolite biosynthesis (*P* adjust < 0.05) (**Figure 8A**, **Supplementary Table 3**). Specifically, the metabolites were involved in linoleic acid metabolism, alpha-linolenic acid metabolism, cutin, suberine and wax biosynthesis, plant hormone signal transduction, biosynthesis of unsaturated fatty acids, flavone and flavonol biosynthesis, arachidonic acid metabolism and monoterpenoid biosynthesis.

Further analysis of the module’s internal connectivity identified the top 30 hub metabolites, which constitute the probable regulatory core of this network (**Figure 8B**, **Supplementary Table 4**). These hub metabolites included pivotal JA precursors and oxylipins, such as 9-F1-phytoprostane and 12-oxo-2,3-dinor-10,15-phytodienoic acid, which are known to connect lipid peroxidation to defense gene expression (Wasternack and Song, 2017). We also identified a suite of direct antimicrobial compounds, including sesquiterpenes like zedoarondiol and dehydrocurdione (Tholl, 2015), alongside with protective antioxidants such as the flavonoid glycoside isorhamnetin 3-O-[β-D-glucopyranosyl-(1→2)-α-L-rhamnopyranoside] (Falcone Ferreyra et al., 2012). The hub metabolites also included volatile aldehydes like 2,4-undecadienal that may function in plant-environment interactions (Dudareva et al., 2013). Therefore, these metabolites that exhibited high connectivity within the turquoise module, might play a central regulatory role in seedling development; furthermore, considering that these metabolites are involved in defense responses, oxidative stress responses, and plant hormone signal transduction, these pathways may contribute extensively in rice seedling development.

### Integrated metabolome and transcriptome analysis of CS2022 and HXR450 in seedling development following direct seeding

To further investigate the mechanism underlying the difference in seedling vigor between the two varieties, we first compared the accumulation levels of the 30 key metabolites in the turquoise module between CS2022 and HXR450. However, no significant difference was observed (**Supplementary Figure 3**). We then performed transcriptome sequencing on 5 DAS seedlings following direct seeding of both varieties and conducted a multi-omics integrated analysis. The PCA results showed that PC1 and PC2 explained 41.06% and 23.08% expression variation of the two samples, respectively (**Supplementary Figure 4A**). Hierarchical clustering heatmaps showed high correlation (R^2^>0.858) between the biological replicates of the two varietal samples (**Supplementary Figure 4B**). This indicated that the data are reliable and can be used for further analysis. A total of 1077 DEGs were identified (|log_2_(FC)| ≥ 1, FDR < 0.05). Among them, 448 and 629 genes were respectively up-regulated and down-regulated in CS2022 seedlings (**Supplementary Figure 5A**, **Supplementary Table 8**).

**Figure 9:**
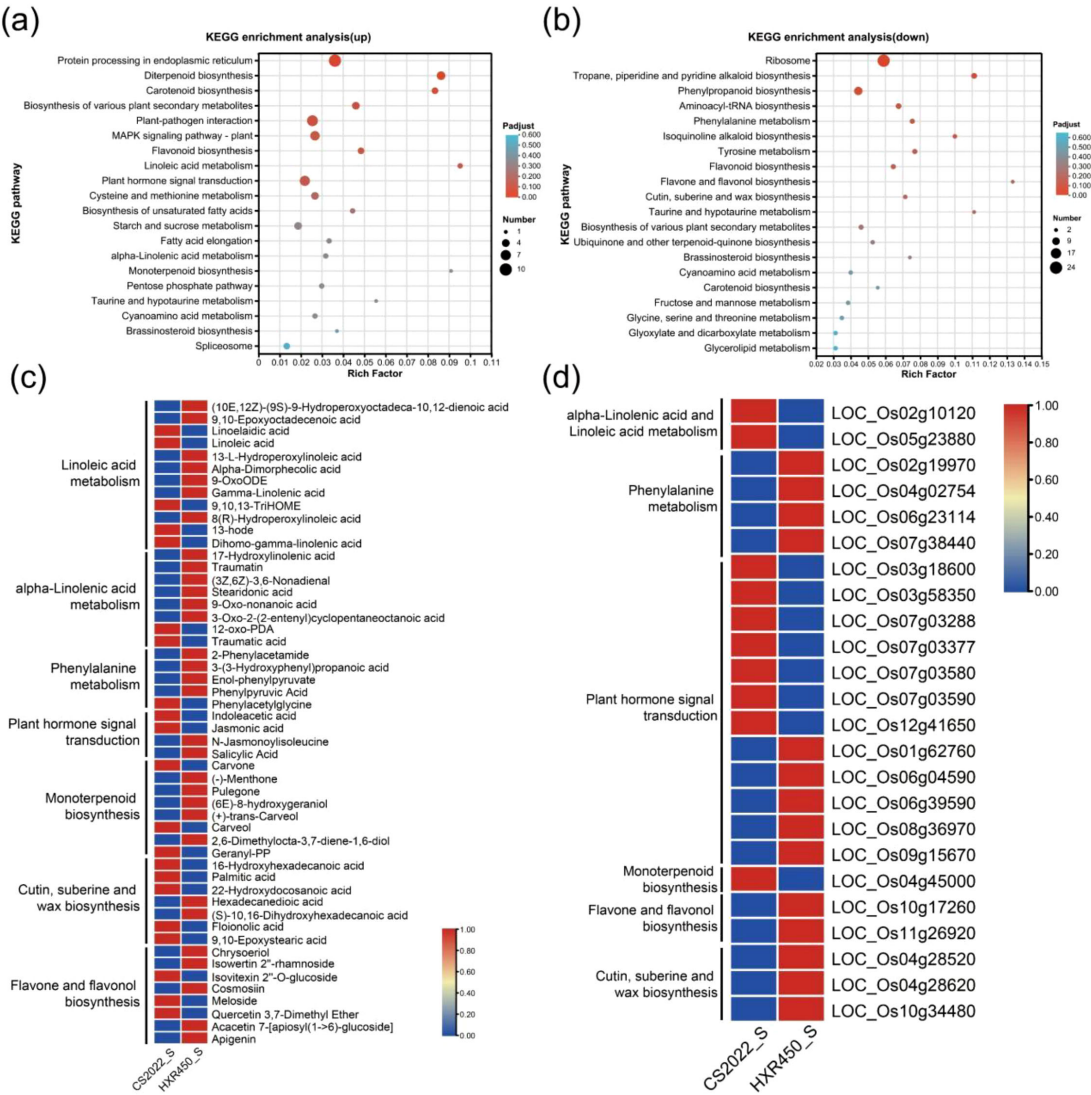
Identification of differentially regulated pathways between CS2022 and HXR450 seedlings by transcriptome-metabolome integrated analysis. **(A, B)** The KEGG enrichment analysis of up-regulated DEGs **(A)** and down-regulated DEGs **(B)**. **(C, D)** Metabolite abundance **(C)** and gene expression **(D)** in pathways enriched by both turquoise module and DEGs. The left column displays the pathway names. Data has been normalized. Red color indicates higher abundance, while blue indicates relatively lower abundance.

GO enrichment analysis of DEGs between the two varieties revealed that up-regulated DEGs were significantly enriched in biological processes such as stress responses (e.g., responses to heat and oxidative stress), terpenoid biosynthesis, redox enzyme activity, and protein processing in the endoplasmic reticulum (**Supplementary Figures 5B, C**, **Supplementary Table 9**). KEGG pathway analysis indicated that the up-regulated DEGs were enriched in pathways including diterpene biosynthesis, carotenoid biosynthesis, plant-pathogen interactions, MAPK signaling, plant hormone signaling transduction, linoleic acid metabolism and α-linolenic acid metabolism (**Figure 9A**, **Supplementary Table 10**). These pathways overlapped well with those enriched in metabolites within the turquoise module (**Figure 8A**). Meanwhile, down-regulated DEGs were significantly enriched in pathways including cell wall organization, ribosome function, phenylpropanoid biosynthesis, flavonoid and flavanol biosynthesis, as well as cutin, suberine, and wax biosynthesis (**Figure 9B**). Notably, the flavonoid and flavanol biosynthesis pathways, along with the cutin, suberine, and wax biosynthesis pathways, were also enriched in the metabolome turquoise module (**Figure 8A**).Therefore, the multi-omics integrated analysis indicated that although the hub metabolites levels are similar between the two cultivars, CS2022 may keep its superior seedling vigor by modulating gene expression in related pathways, thus to optimize resource allocation while maintaining metabolite homeostasis in these pathways.

### Potential mechanism underlying difference in seedling vigor between CS2022 and HXR450

The above analysis makes it clear that plant hormone signaling, linoleic acid metabolism, α-linolenic acid metabolism, phenylalanine metabolism, monoterpenoid metabolism, flavonoid and flavanol biosynthesis, cuticle, suberin, and wax biosynthesis were significantly enriched in both the turquoise module and DEGs (**Figures 8A**, **9A, B**). We further specified the metabolites (**Figure 9C**) and genes (**Figure 9D**) enriched in these pathways, it was revealed that most of these pathways are associated with hormone synthesis and signaling transduction. For example, linoleic acid and α-linolenic acid metabolism are key sources of JA synthesis, and phenylalanine metabolism is linked to auxin synthesis. Given the central role of phytohormonal regulation, we therefore prioritized an integrated mapping of these metabolites and genes onto the hormone biosynthesis and signaling pathways (**Supplementary Figure 6**). In the JA related pathway, biosynthesis was enhanced, as evidenced by the up-regulation of two lipoxygenase genes *OsLOX7* (*LOC_Os05g23880*) and *OsLOX1* (*LOC_Os02g10120*) (**Figure 9D**), and by elevated levels of the intermediate 12-oxo-PDA and JA (**Figure 9C**). However, there was a marked decrease in N-Jasmonoylisoleucine (JA-Ile), the bioactive form of JA (**Figure 9C**). To assess whether reduced JA-Ile in CS2022 reflects suppressed signaling, we examined the expression of key JA pathway genes, including *OsCOI1a*, *OsCOI1b*, *OsJAZ1*, *OsMYC2*, and primary JA-Ile synthase *OsJAR1*. No significant differential expression was detected for any of these genes (**Supplementary Table 8**). This indicates that the JA signaling machinery remains transcriptionally intact, and the reduced JA-Ile level is unlikely attributable to suppressed expression of its biosynthetic components. Concurrently, auxin signaling was activated, characterized by increased auxin abundance and diffe3zrential regulation of Aux/IAA genes, for example, *OsIAA23* (*LOC_Os06g39590*) was down-regulated, while *OsIAA14* (*LOC_Os03g58350*) was up-regulated (**Figure 9D**). Despite the decreased content of SA, downstream defense was primed as evidenced by the concerted up-regulation of four pathogenesis-related (PR) genes (*LOC_Os07g03288*, *LOC_Os07g03580*, *LOC_Os07g03377*, *LOC_Os07g03590*). We validated the expression levels of these eight genes via qRT-PCR, and the results showed consistency with the trends observed in the transcriptome (**Supplementary Figure 7**). This hormonal configuration demonstrates an optimized balance between growth and defense, promoting rapid seedling establishment in CS2022.

## Discussion

High emergence vigor is an important characteristic of suitable direct seeding rice varieties (Mahender et al., 2015; Li et al., 2023). Previous studies have demonstrated that long mesocotyls enable seedlings to emerge quickly from deep soil and have primarily deciphered the genetic mechanisms underlying the superior emergence vigor (Lv et al., 2021; Lyu et al., 2024). However, there remains a lack of understanding regarding the dynamic changes in metabolites during rice direct seeding. In this study, we employed an integrated metabolomic and transcriptomic approach to dissect the molecular basis of the high emergence vigor observed in the elite direct-seeding variety CS2022, in comparison to the control variety HXR450 (**Figures 1A–F**, **Supplementary Figures 1A–C**). Our study reveals that the superior performance of CS2022 in germination is not driven by a single gene, but rather by a systemic optimization of hormonal homeostasis, metabolic sensitivity, and growth-defense trade-offs.

### Physiological validation of hub metabolites: cytokinins and amino acids as drivers of germination

WGCNA positioned cytokinins (specifically zeatin and *cis*-zeatin-9-N-glucoside) and amino acids (e.g., L-lysine) as central “hub” metabolites in the germination module of CS2022 (**Figures 4C, D**, **5**). The high accumulation of these compounds is tightly correlated with the germinating vigor, moreover, our physiological validation using *trans*-zeatin, the primary active form of zeatin, and L-lysine provided causal evidence further support their role in germination vigor. Crucially, CS2022 displayed a “heightened sensitivity” phenotype: it achieved maximal germination promotion at concentrations 10-fold lower than HXR450. Thus, CS2022 might possesses a more responsive signaling machinery. Cytokinins are known to antagonize ABA by down-regulating *ABI5* expression (Wang et al., 2011). A lower threshold for cytokinin response allows CS2022 to break dormancy and initiate cell division more efficiently. Similarly, the sensitivity to L-lysine supports the hypothesis that specific amino acids may act not just as nutrients, but as signaling cues to trigger metabolic reactivation (Xiao et al., 2025). This high-sensitivity strategy likely enables CS2022 to rapidly initiate germination upon imbibition, providing a vital competitive advantage.

### Hormonal homeostasis: the GA_20_ reservoir and ABA deactivation

This superior performance of CS2022 is also underpinned by a GA/ABA regulatory backbone (Hazman et al., 2019). While bioactive GA_1_ levels were below the detection threshold, likely due to rapid turnover during the early imbibition phase, we observed a striking accumulation of its immediate precursor, GA_20_, in CS2022 germinating seeds (**Figure 6A**). In rice, the synthesis of GA_1_ by GA20-oxidase (encoded by *SD1*) is a critical rate-limiting checkpoint for vigor and elongation (Sasaki et al., 2002; Spielmeyer et al., 2002). At the same time, our targeted LC-MS/MS assays revealed that CS2022 converts more free ABA into the inactive ABA-glucosyl ester (ABA-GE) form. The significantly elevated GA_20_/ABA ratio in CS2022 (**Figure 6E**) suggests that this variety maintains a larger substrate pool poised for rapid conversion to bioactive GAs upon imbibition. This mechanism aligns with findings by Du et al (Du et al., 2015), who demonstrated that upregulation of the GA_20_/ABA ratio is a hallmark of dormancy release in rice. Thus, CS2022 overcomes the ABA-imposed inhibition not just by lowering ABA, but by maintaining a higher biosynthetic pressure (GA_20_ flux) to activate downstream α-amylase activity (**Figure 6F**) (Shu et al., 2016).

### Optimizing the growth-defense trade-off during emergence

At the seedling establishment stage, a key challenge is balancing rapid elongation with defense against soil pathogens (Ma et al., 2020). Our multi-omics integration reveals a distinct pattern in CS2022, whereby the upregulation of JA biosynthesis transcripts is accompanied by relatively low accumulation of the bioactive hormone JA-Ile. In plants, high JA-Ile levels typically trigger JAZ degradation, leading to growth inhibition, known as the classic “growth-defense trade-off” (Thines et al., 2007; Johnson et al., 2023). Therefore, the observed transcript-metabolite decoupling may represent a potential “signal buffering” mechanism. We hypothesize that by limiting the accumulation of bioactive JA-Ile despite high precursor gene expression, CS2022 could prioritize auxin-mediated rapid elongation to escape deep soil, potentially avoiding the growth penalties associated with excessive defense activation. As previously reported (Chung et al., 2010; Zhai et al., 2013; Xiao et al., 2025), the underlying mechanisms behind this decoupling phenomenon may involve regulation at multiple levels, including post-transcriptional, translational, and post-translational regulation.

However, as this study is based on steady-state omics data, further functional verification is required for confirmation, such as quantifying JAZ protein stability or pathogen response kinetics.

### Lipid remodeling: an adaptive strategy for hypoxia

Finally, the deep-sowing environment often imposes hypoxic stress (Ma et al., 2020). The enrichment of sphingolipids and cuticular wax biosynthesis pathways in turquoise module aligns with adaptations to hypoxic environment (**Figure 6A**). Sphingolipids are essential for maintaining plasma membrane integrity and ion homeostasis under low-oxygen stress (Vishal and Kumar, 2018). Concurrently, the synthesis of cuticular waxes and very-long-chain fatty acids facilitates the formation of hydrophobic barriers on the coleoptile surface, a known mechanism in flood-tolerant plants to enhance gas exchange underwater (Shi et al., 2025). Therefore, the lipid remodeling observed in CS2022 is likely to be a proactive adaptation to ensure seedling survival in waterlogged, hypoxic conditions, rather than just a developmental by-product. Although these metabolic associations align with hypoxia adaptation signatures, they are currently correlative and warrant causal validation.

## Conclusion

In summary, CS2022 exhibits superior emergence vigor through a multi-layered strategy: (1) enhanced sensitivity to cytokinins and amino acids for rapid metabolic activation; (2) hormonal homeostasis via GA precursor accumulation and ABA deactivation; (3) suppression of defense-induced growth arrest; and (4) lipid remodeling for hypoxia tolerance. These findings outline a correlative network and pinpoint metabolic targets for breeding high-vigor rice. However, conclusions are drawn from only two contrasting varieties, and some responses may be genotype-specific. Future studies using RIL or natural populations are needed to validate these pathways and dissect the underlying QTLs via GWAS.

## Data Availability

The data presented in the study are deposited in the NCBI GEO repository (accession number: GSE322568) and MetaboLights repository (accession number: MTBLS13952).
