# Supplementary material for: Integrated metabolome and transcriptome profiling demonstrates dynamic regulatory roles of hormones in direct-seeding rice
Source: Front Plant Sci. 2026 Mar 10;17:1767519. doi: 10.3389/fpls.2026.1767519 (PMC13008863; doi:10.3389/fpls.2026.1767519)
Supplement: Supplementary file 2 [file DataSheet1.docx]

**
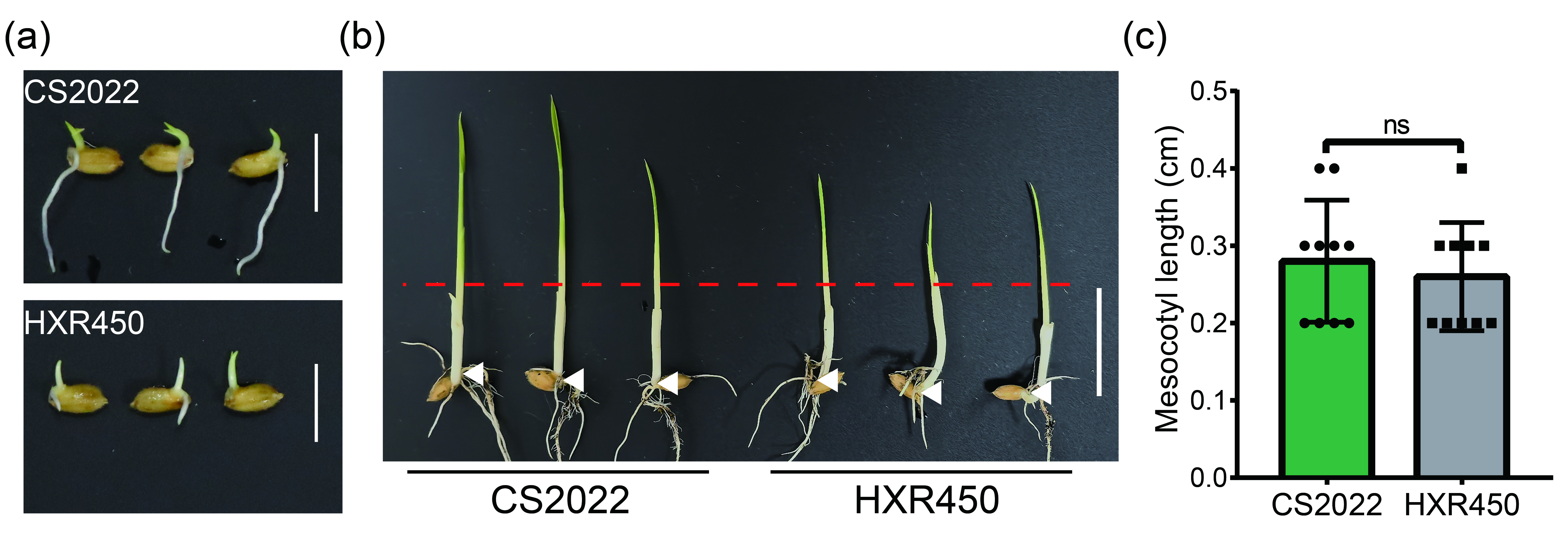
Supplementary figure 1. Comparison of seed germination and seedling emergence of CS2022 and HXR450**

(a) Shoot and root development of CS2022 and HXR450 seeds after two days of germination induction. Scale bar = 1 cm. (b) The image of seedlings of CS2022 and HXR450 5 DAS. The arrowheads point to the coleoptile nodes. The red dotted line indicates the position of the soil surface. The scale bar is 2 cm. (c) Statistical comparison of the mesocotyl length of CS2022 and HXR450 5 DAS. Values are means ± SD, n = 10. The dots show each value. Data were analyzed by Student’s *t* tests, Ns means not significant.

**
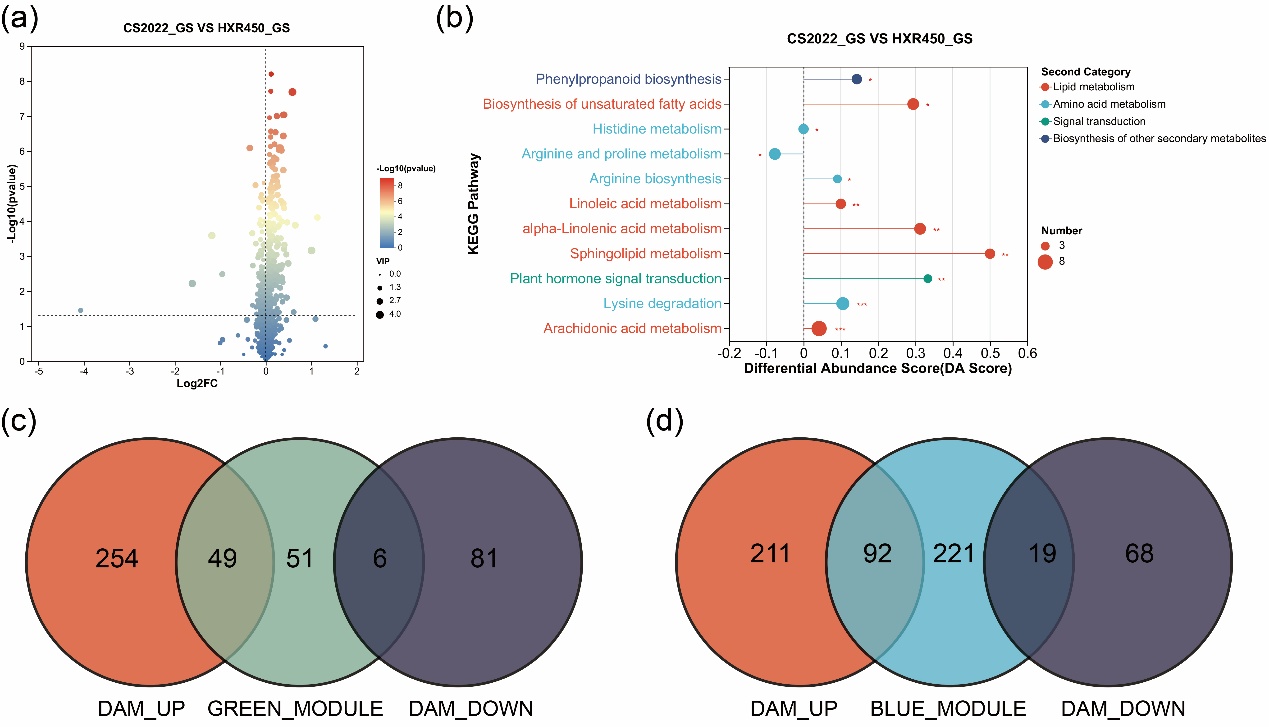
Supplementary figure 2. Comparison of metabolite levels of CS2022 and HXR450 germinating seeds**

(a) Volcano plot of differentially accumulated metabolites in CS2022 and HXR450 germinated seeds. The horizontal coordinate is the value of the fold change of the difference in metabolite content between the two groups, and the vertical coordinate is the value of the statistical test for the difference in the change in metabolite content. The values of the horizontal and vertical coordinates are logarithm zed. Each point in the graph represents a specific metabolite, and the size of the point indicates the VIP value. (b) Differential abundance (DA) scores for CS2022 and HXR450 germinating seed metabolites. Different metabolic pathways are indicated by different color fonts. 1 and -1 represent that all identified metabolites in the pathway are up-regulated and down-regulated, respectively. The size of the dot represents the number of metabolites. (c-d) Venn diagram of metabolites in germination-related modules, up-regulated DAMs and down-regulated DAMs.

**
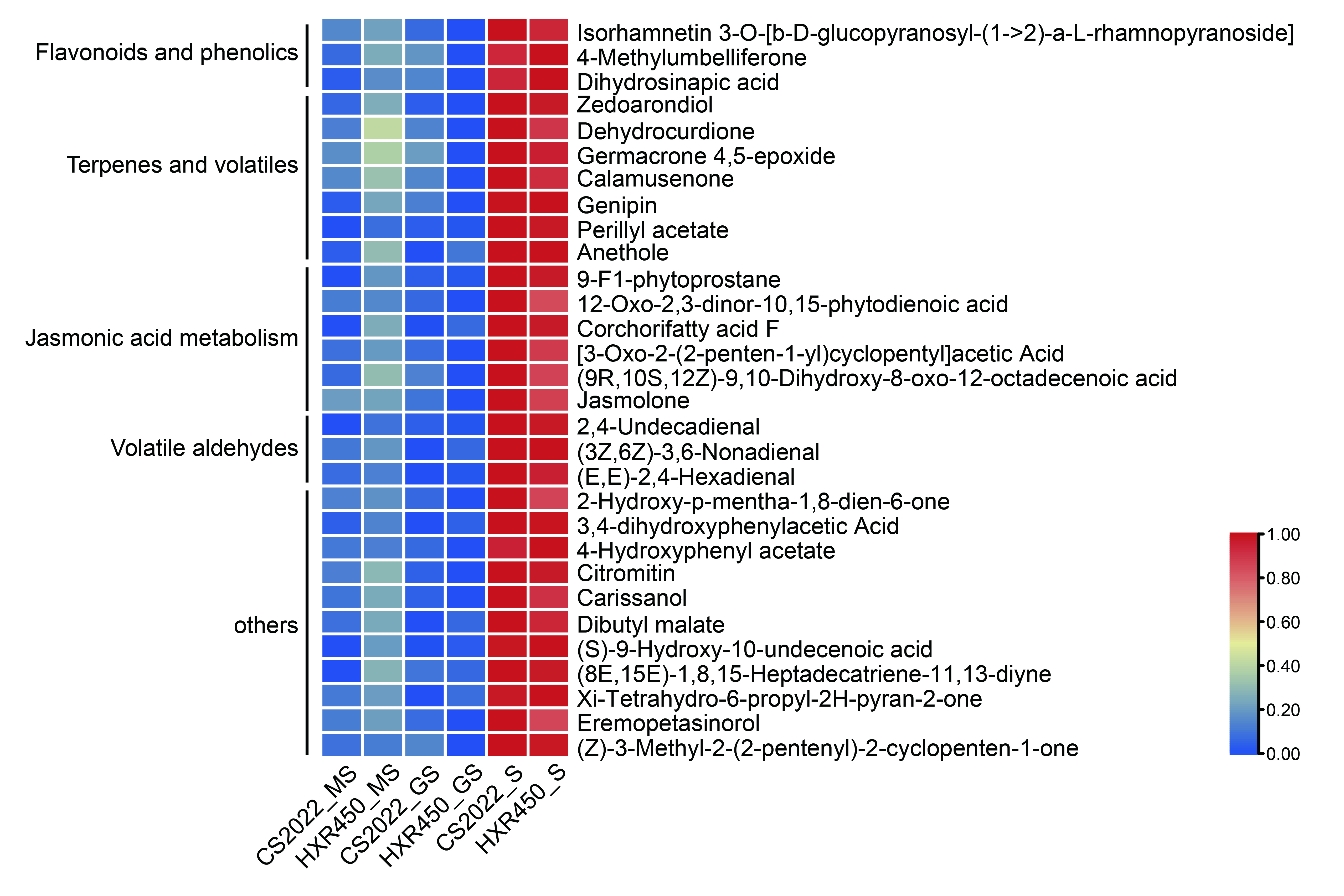
Supplementary figure 3. Heatmap of hub metabolites of CS2022 and HXR450 in the turquoise module**

Metabolites are annotated according to their pathways. Data has been normalized.


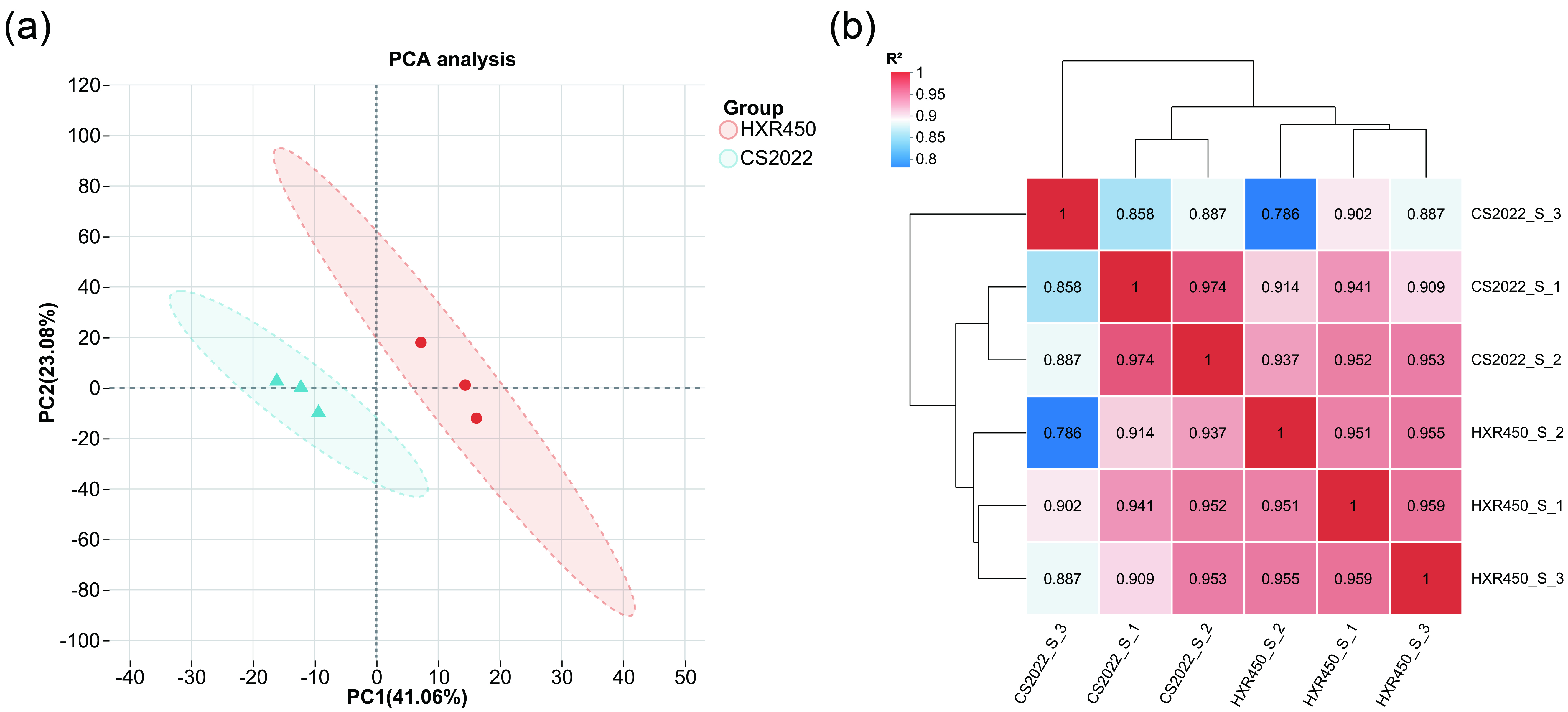
**Supplementary figure 4. Principal component analysis (PCA) and correlation heatmap of transcriptomic profiling of CS2022 and HXR450 seedlings**

(a, b) The PCA results (a) showed significant separation between the two varieties, and the correlation heat map (b) results indicated a high degree of association between the replicates.

**
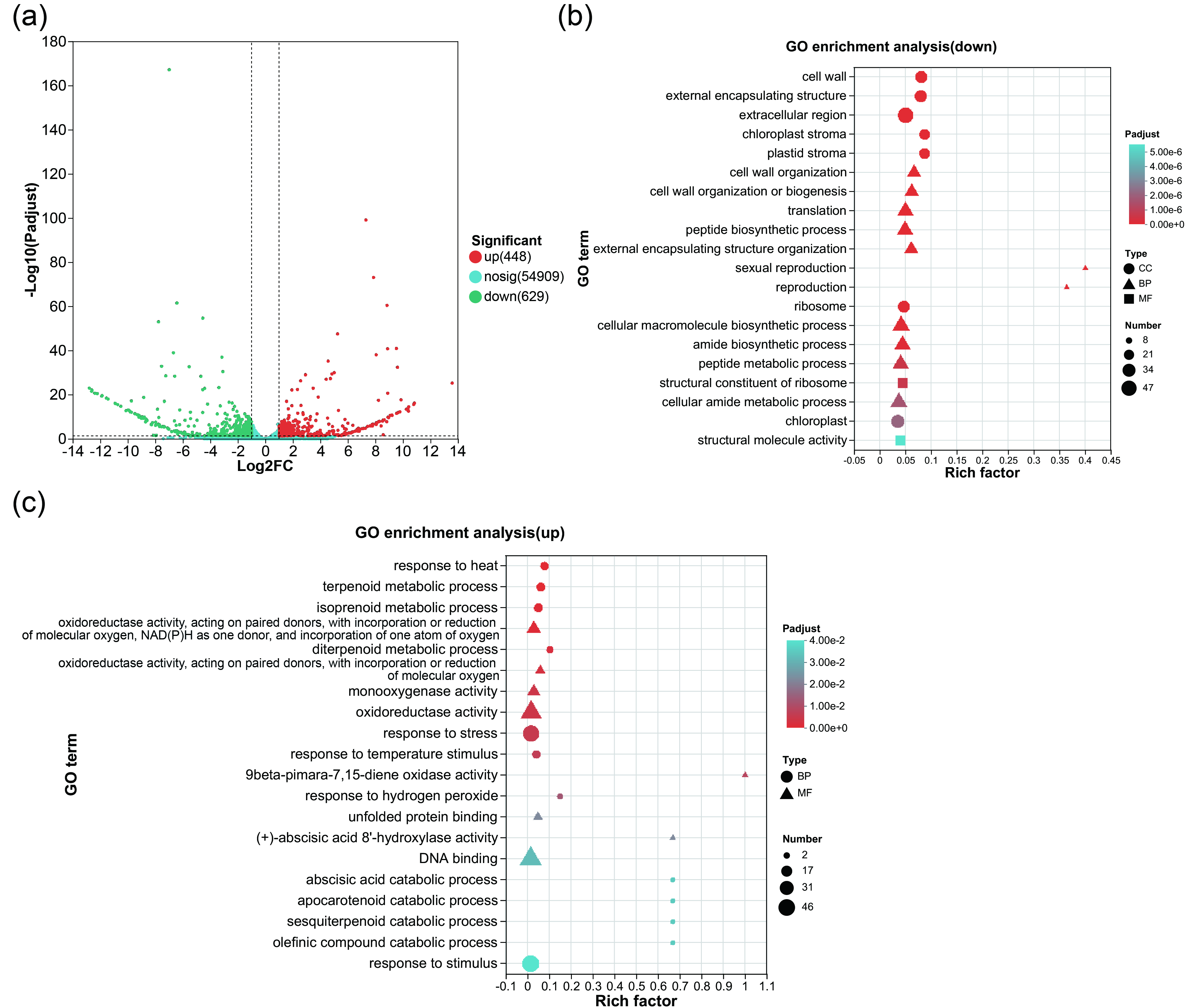
Supplementary figure 5. Volcano plot of DEGs in CS2022 and HXR450 seedlings and GO enrichment analysis of DEGs**

1. Volcano plot of DEGs in CS2022 and HXR450 seedlings. The DEG screening criteria is *P* adjust ≤ 0.05 and |FoldChange| ≥ 2. (b-c) GO enrichment analysis of down-regulated (b) and up-regulated (c) DEGs.

**
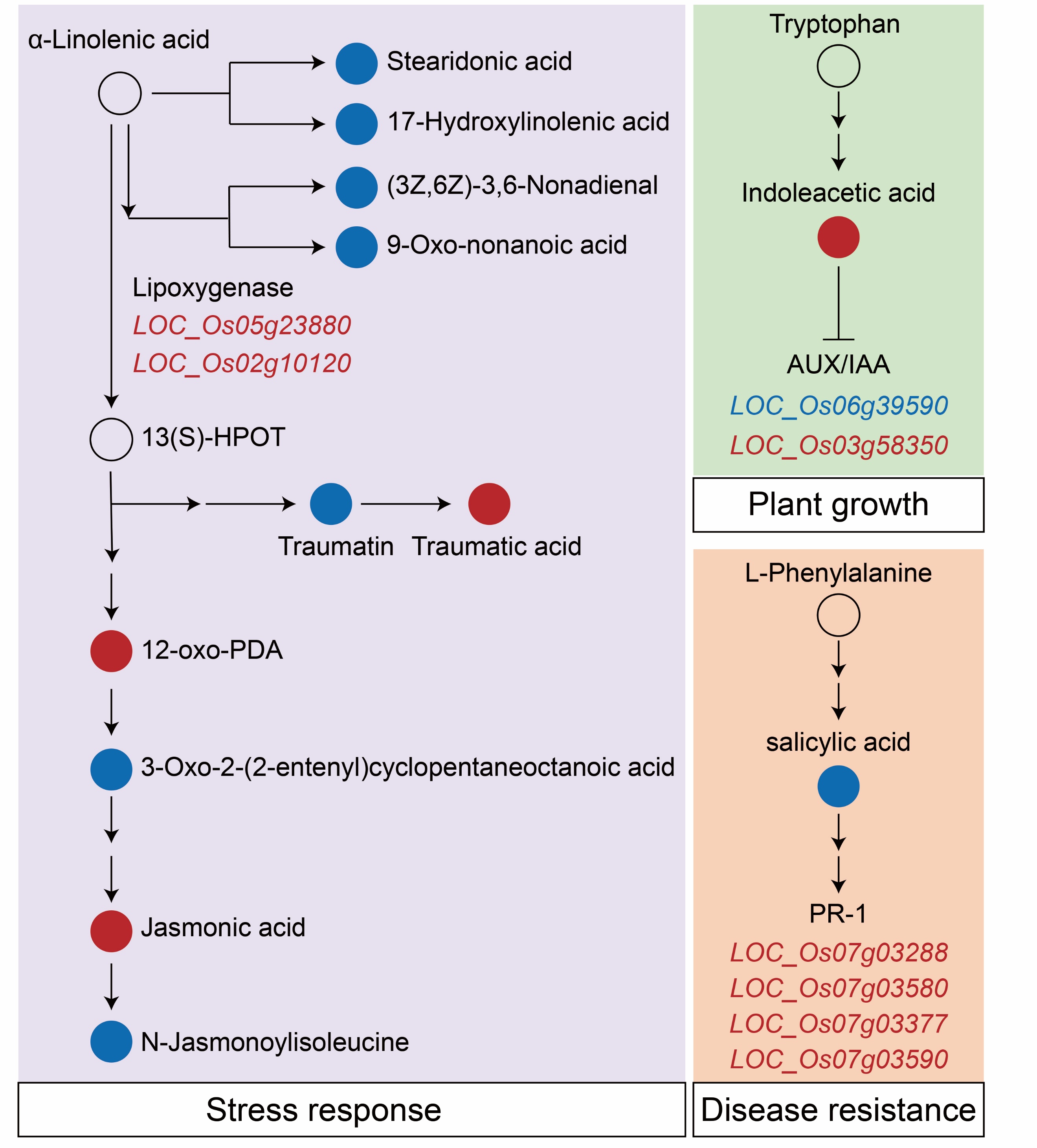
Supplementary figure 6. Plant hormones synergistically regulate seedling establishment**

Co-expression analysis of metabolites and genes related to plant hormone synthesis and signaling transduction pathways. Dots represent metabolites. Red dots indicate metabolites whose abundance were up-regulated in CS2022 seedlings relative to HXR450, while blue dots indicate down-regulation. Red gene symbols denote genes whose expression is up-regulated in CS2022 seedlings, and blue symbols denote down-regulated genes.


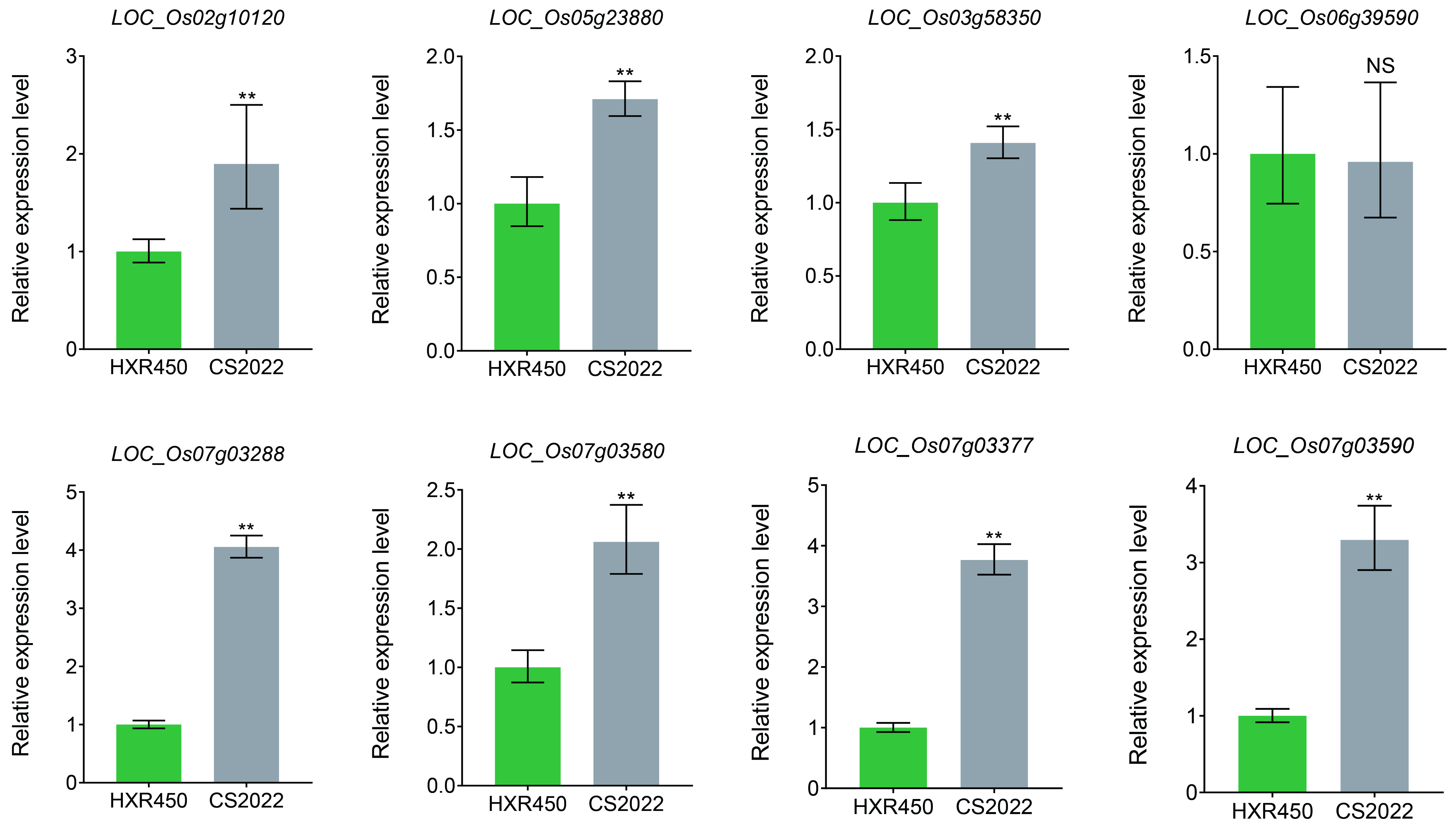
**Supplementary figure 7. qRT-PCR validation of the selected 8 gene expression levels**

Data are mean ± SD (n = 3). Student’s t-test was carried out (***P* < 0.01).
